# Symmetry-based approach to oligostilbenoids: Rapid entry to viniferifuran, shoreaphenol, malibatol A, and diptoindonesin G

**DOI:** 10.3762/bjoc.12.266

**Published:** 2016-12-12

**Authors:** Youngeun Jung, Dileep Kumar Singh, Ikyon Kim

**Affiliations:** 1 College of Pharmacy and Yonsei Institute of Pharmaceutical Sciences, Yonsei University, 85 Songdogwahak-ro, Yeonsu-gu, Incheon 21983, Republic of Korea

**Keywords:** anticancer agent, iodocyclization, natural product, oligostilbenoids, Pd-catalyzed coupling

## Abstract

The recognition of the local symmetric image within benzofuran-based natural oligostilbenoids guided us to design a modular synthetic approach to these molecules by utilizing a three-step sequence consisting of Sonogashira coupling, iodocyclization, and Suzuki coupling. During our synthesis, the relative reactivities of ester, aldehyde, and alkoxy groups on the same aryl ring toward the neighboring alkyne in the iodine-mediated cyclization reactions were explored. Starting from the symmetrical 3,5-dimethoxybenzyl alcohol, this route allowed rapid access to 2,3-diarylbenzofuran, a key intermediate to several oligostilbenoid natural products, in good overall yields.

## Introduction

**Figure 1:**
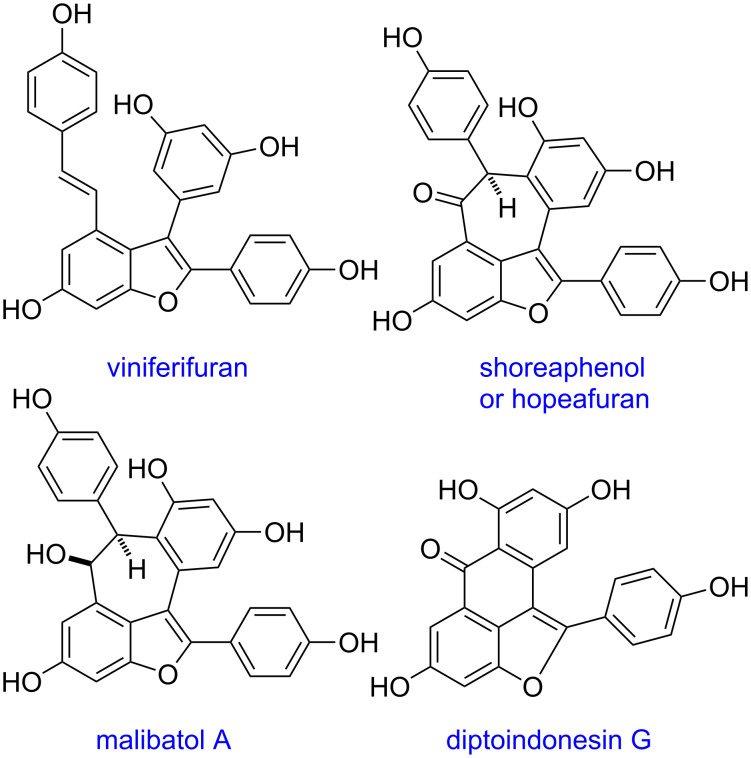
Natural oligostilbenoids.

Oligostilbenoids constitute a family of natural products with various biological functions (Figure 1). Monomeric stilbene units are interconnected in a number of ways to lead to complex structures [1–3]. Despite a long history of isolation and biological studies of these natural products, relatively little attention has been paid by the synthetic community to chemical synthesis of polyphenolic oligostilbenoids. Most synthetic works on these unique natural products have recently appeared in the literature [4–10].

**Scheme 1:**
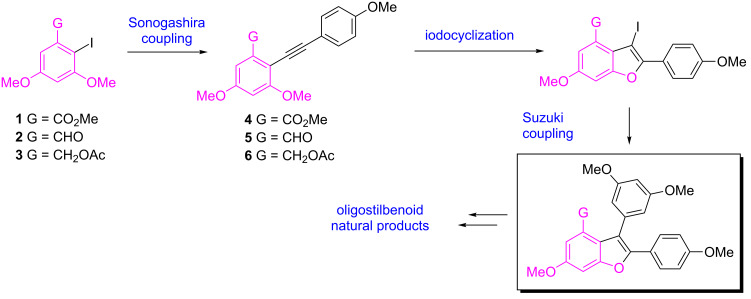
Synthetic plan.

In connection with our research on benzofurans [11–12], our laboratory has been involved in the synthesis of these benzofuran-containing natural products for the last several years [13–15]. For example, we have reported a concise total synthesis of diptoindonesin G, a potent cytotoxic and immunosuppressant agent [16–17], by using a highly efficient domino cyclodehydration/intramolecular Friedel–Crafts acylation/regioselective demethylation sequence as a key transformation. Very recently, a dual functional role of diptoindonesin G in modulating α and β estrogen receptors (ER) has been discovered, thereby suggesting it as a promising drug lead for the treatment of breast cancer [18]. Our continuing interest in this area led us to design an alternative approach to oligostilbenoids. As shown in Scheme 1, our idea stemmed from recognition of the symmetry element [19] of the target molecules. We expected that the key intermediate (inset box of Scheme 1) could be constructed from the monoiodo compounds **1**, **2**, or **3** through a sequence involving Sonogashira coupling, iodocyclization [20–26], and Suzuki coupling. As the starting materials (**1**, **2**, and **3**) were readily available from the corresponding *C*2 symmetric precursors via monoiodination, we decided to evaluate this route. In particular, we wondered what functional group as a G moiety would be appropriate for the successful iodine-mediated cyclization. Ester, aldehyde, and alkoxy groups have been used as nucleophiles of iodocyclization for the syntheses of a number of heterocycles, respectively [27–30]. Although Larock’s work on relative reactivity of these functional groups toward alkyne during electrophilic cyclization has been reported [31], the study with the substrates having these nucleophiles on the same aromatic ring has not been disclosed, to the best of our knowledge. Here we wish to describe our results.

## Results and Discussion

**Scheme 2:**
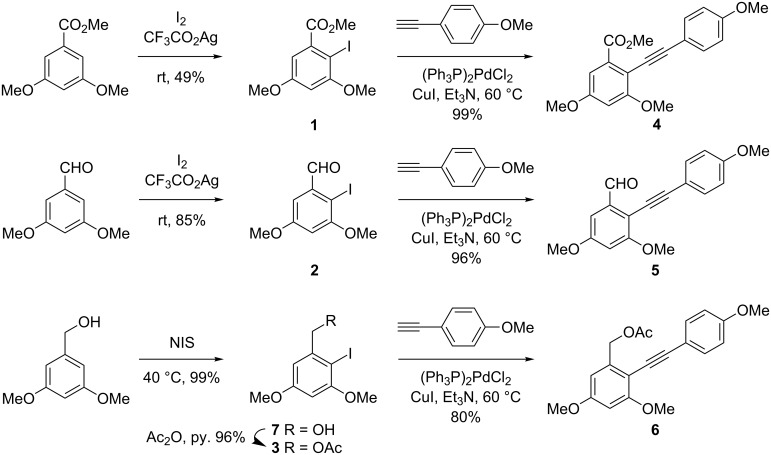
Synthesis of **4**, **5**, and **6**.

By following the known procedures, monoiodination of the commercially available methyl 3,5-dimethoxybenzoate, 3,5-dimethoxybenzaldehyde, and 3,5-dimethoxybenzyl alcohol under the influence of either I_2_/silver trifluoroacetate or *N*-iodosuccinimide afforded **1** [32–34], **2** [35], and **7** [36–37], respectively (Scheme 2). The hydroxy group of **7** was protected as an acetate, providing **3** in 96% yield. Sonogashira coupling of the resulting iodides **1**, **2**, and **3** with alkynylanisole proceeded without any event to give the corresponding alkynes, **4**, **5**, and **6**, setting the stage for iodocyclization.

**Scheme 3:**
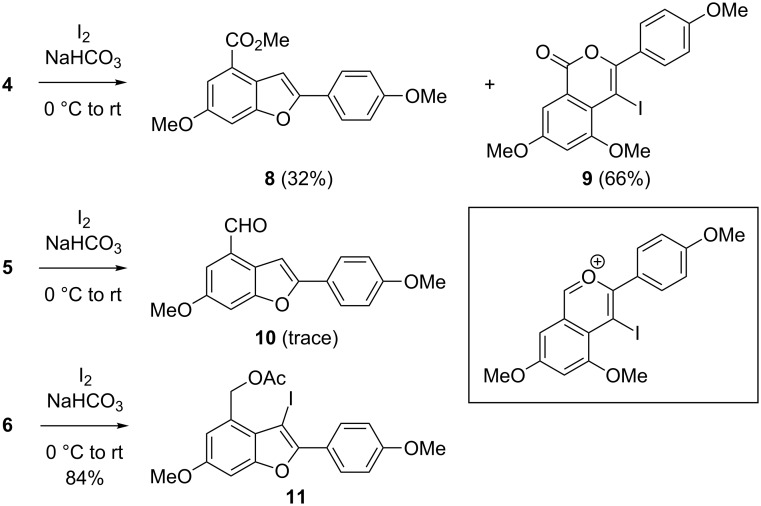
Iodocyclization.

**Figure 2:**
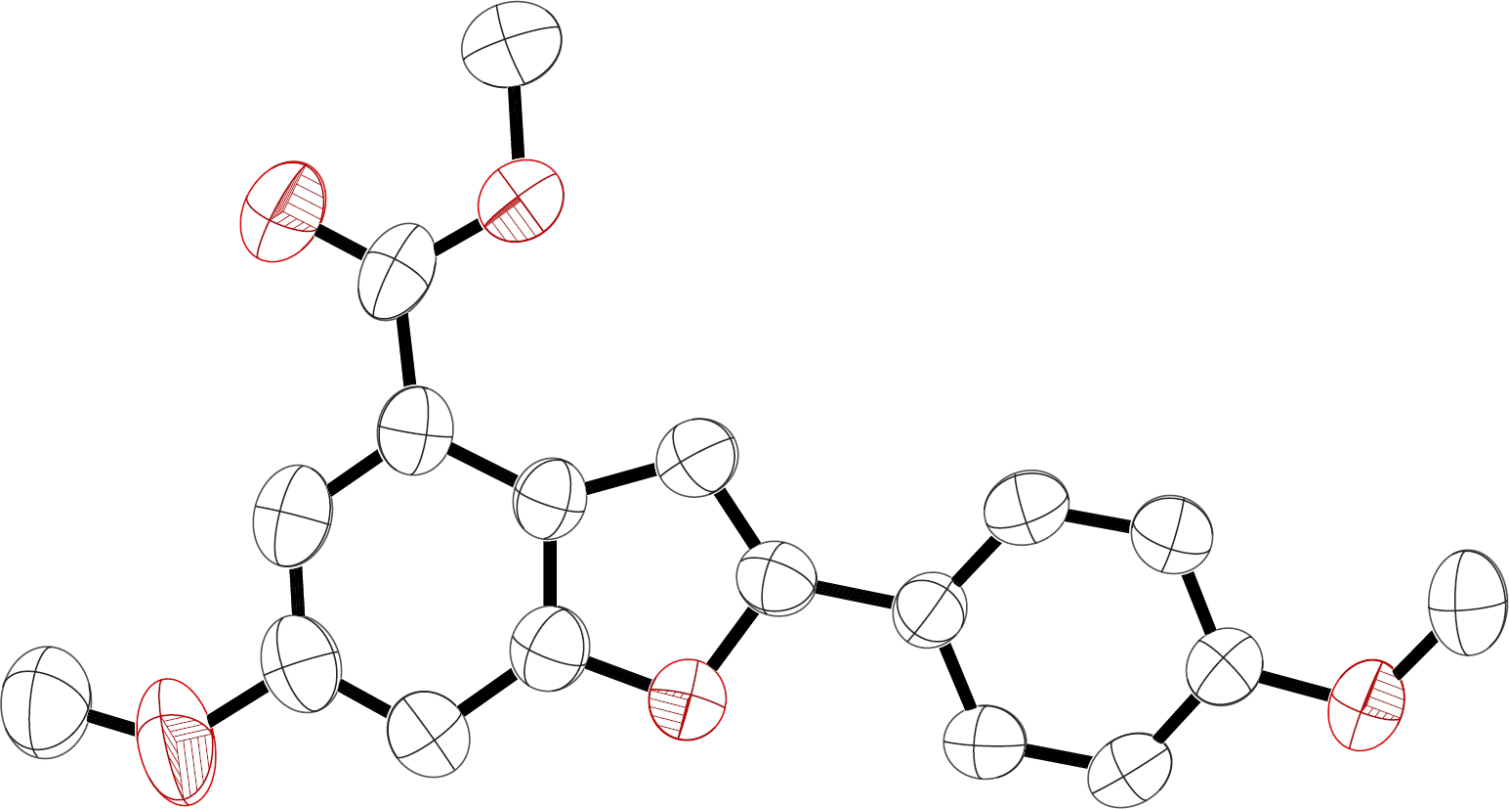
Crystal structure of **8**.

When **4** was exposed to I_2_ and NaHCO_3_ in CH_2_Cl_2_, two isolable products were obtained (Scheme 3). Surprisingly, **8** was isolated in 32% yield presumably as a consequence of HI-promoted cyclization even in the presence of excess base. The structure of **8** was confirmed by X-ray crystallographic analysis (Figure 2) [38]. The other major product **9**, less polar than **8**, resulted from 6-*endo*-dig iodocyclization. Obviously, the ester moiety in **4** was competitively involved in the iodine-mediated electrophilic cyclization. Only a trace amount of **10** was isolated upon subjection of **5** to the same reaction conditions. We suspected that the aldehyde in **5** also acted as a nucleophile to furnish unstable oxocarbenium species (inset box of Scheme 3) as a major product which decomposed eventually. On the other hand, **6** was successfully converted to the desired 3-iodobenzofuran **11** in good yield. These results led us conclude that either ester or aldehyde groups perform as better nucleophiles than an alkoxy group in the iodocyclization.

**Scheme 4:**
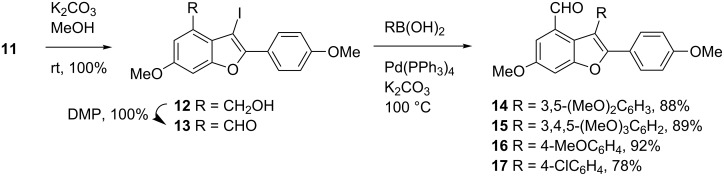
Synthesis of **14**.

Having secured gram quantities of 3-iodobenzofuran **11** in hand, our next task was to elaborate the conversion of **11** to 2,3-diarylbenzofurans (Scheme 4). To this end, **11** was first transformed to aldehyde **13** via **12** through a two-step sequence consisting of deacetylation and Dess–Martin oxidation [39]. To our delight, the subsequent Suzuki cross-coupling of **13** with 3,5-dimethoxyphenylboronic acid under the reaction conditions analogous as described before [26] proceeded well and furnished **14** in 88% yield. This intermediate was used for our previous syntheses of permethylated analogues of viniferifuran, malibatiol A, and shoreaphenol [13]. Under similar reaction conditions, several other arylboronic acids reacted with **13** to give the corresponding products in good yields, demonstrating the general usefulness of this route for the synthesis of a range of structural analogues at a late stage.

**Scheme 5:**
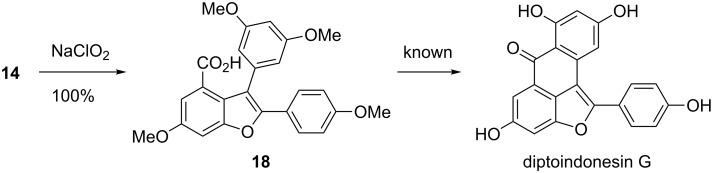
Synthesis of **18**.

The direct Friedel–Crafts type intramolecular cyclization of **14** induced by BCl_3_ was attempted but a complex mixture was observed. Thus, oxidation of the aldehyde in **14** to carboxylic acid was carried out. Pinnick oxidation [40–42] of **14** went smoothly to give **18**, an intermediate previously employed for the synthesis of diptoindonesin G (Scheme 5) [43–44].

## Conclusion

In summary, we have established a highly scalable and flexible synthetic route to several benzofuran-containing oligostilbenoid natural products by relying on a symmetry-breaking strategy from 3,5-dimethoxybenzyl alcohol. The relative reactivity of ester, aldehyde, and methoxy moieties toward the neighboring alkyne in the course of iodocyclization was investigated, revealing that neither ester nor aldehyde was compatible under these conditions to reach the desired product. The versatile key intermediates for the syntheses of several oligostilbenoids such as viniferifuran, shoreaphenol, malibatol A, and diptoindonesin G were rapidly accessed in a highly efficient manner, allowing for large-scale preparations of the target natural products as well as unnatural analogues.

## Supporting Information

File 1 Experimental procedures, compound characterization data, and ^1^H and ^13^C NMR spectra of synthesized compounds.

File 2 Chemical information file of compound **8**.
