# Supplementary material for: Symmetry-based approach to oligostilbenoids: Rapid entry to viniferifuran, shoreaphenol, malibatol A, and diptoindonesin G
Source: Beilstein J Org Chem. 2016 Dec 12;12:2689–93. doi: 10.3762/bjoc.12.266 (PMC5238531; doi:10.3762/bjoc.12.266)
Supplement: File 1 — Experimental procedures, compound characterization data, and 1H and 13C NMR spectra of synthesized compounds. [file Beilstein_J_Org_Chem-12-2689-s001.pdf]

Supporting Information

for

Symmetry-based approach to oligostilbenoids: Rapid entry to viniferifuran, shoreaphenol, malibatol A, and diptoindonesin G

Youngeun Jung[‡], Dileep Kumar Singh[‡] and Ikyon Kim*[§]

Address: College of Pharmacy and Yonsei Institute of Pharmaceutical Sciences, Yonsei University, 85 Songdogwahak-ro, Yeonsu-gu, Incheon 21983, Republic of Korea

*Corresponding author

Email: Ikyon Kim - ikyonkim@yonsei.ac.kr

[§]Tel.: +82 32 749 4515; fax: +82 32 749 4105

[‡]Equal contributors

Experimental procedures, compound characterization data, and ^{1}H and ^{13}C NMR spectra of synthesized compounds

Table of Contents

Experimental procedures and compound characterization data	**S3–S14**
Copies of ^{1}H and ^{13}C NMR spectra of **1**	**S15**
Copies of ^{1}H and ^{13}C NMR spectra of **2**	**S16**
Copies of ^{1}H and ^{13}C NMR spectra of **3**	**S17**
Copies of ^{1}H and ^{13}C NMR spectra of **4**	**S18**
Copies of ^{1}H and ^{13}C NMR spectra of **5**	**S19**

Copies of ^{1}H and ^{13}C NMR spectra of **6** **S20**

Copies of ^{1}H and ^{13}C NMR spectra of **7** **S21**

Copies of ^{1}H and ^{13}C NMR spectra of **8** **S22**

Copies of ^{1}H and ^{13}C NMR spectra of **9** **S23**

Copies of ^{1}H and ^{13}C NMR spectra of **11** **S24**

Copies of ^{1}H and ^{13}C NMR spectra of **12** **S25**

Copies of ^{1}H and ^{13}C NMR spectra of **13** **S26**

Copies of ^{1}H and ^{13}C NMR spectra of **14** **S27**

Copies of ^{1}H and ^{13}C NMR spectra of **15** **S28**

Copies of ^{1}H and ^{13}C NMR spectra of **16** **S29**

Copies of ^{1}H and ^{13}C NMR spectra of **17** **S30**

Copies of ^{1}H and ^{13}C NMR spectra of **18** **S31**

General methods

Unless specified, all reagents and starting materials were purchased from commercial sources and used as received without purification. “Concentrated” refers to the removal of volatile solvents via distillation using a rotary evaporator. “Dried” refers to pouring onto, or passing through, anhydrous magnesium sulfate followed by filtration. Flash chromatography was performed using silica gel (230–400 mesh) with hexanes, ethyl acetate, and dichloromethane as eluent. All reactions were monitored by thin-layer chromatography on 0.25 mm silica plates (F-254) visualizing with UV light. Melting points were measured using a capillary melting point apparatus. ^{1}H and ^{13}C NMR spectra were recorded on 400 MHz NMR spectrometer and were described as chemical shifts, multiplicity (s, singlet; d, doublet; t, triplet; q, quartet; m, multiplet), coupling constant in hertz (Hz), and number of protons. HRMS were measured with electrospray ionization (ESI) and Q-TOF mass analyzer.

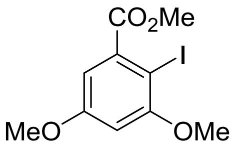

1

Methyl 2-iodo-3,5-dimethoxybenzoate (1). To a stirred solution of methyl 3,5-dimethoxybenzoate (1.0 g, 5.1 mmol) in $CHCl_3$ (25 mL) were added silver trifluoroacetate (1.13 g, 1.0 equiv) and I_2 (1.29 g, 1.0 equiv). After being stirred at rt for 5 h, the reaction mixture was concentrated under reduced pressure to give the crude residue, which was purified by silica gel column chromatography (hexanes/ethyl acetate = 17:3) to give **1**. White solid, mp: 79.9-80.2 °C (771 mg, 49%); **^{1}H NMR** (400 MHz, $CDCl_3$) δ 6.80 (s, 1H), 6.52 (s, 1H), 3.94 (s, 3H), 3.88 (s, 3H), 3.83 (s, 3H); **^{13}C NMR** (100 MHz, $CDCl_3$) δ 168.1, 161.0,

159.5, 139.0, 106.6, 101.4, 75.8, 56.8, 55.8, 52.7; **HRMS** (ESI-QTOF) *m/z* $[M+H]^+$ calcd for $C_{10}H_{12}IO_4$ 322.9775 found 322.9774.

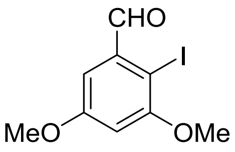

2

2-Iodo-3,5-dimethoxybenzaldehyde (2). To a stirred solution of 3,5-dimethoxybenzaldehyde (500 mg, 3.0 mmol) in CH_2Cl_2 (10 mL) were added silver trifluoroacetate (665 mg, 1.0 equiv) and I_2 (764 mg, 1.0 equiv) in CH_2Cl_2 (2 mL). After being stirred at rt for 5 h, the reaction mixture was concentrated under reduced pressure to give the crude residue, which was purified by silica gel column chromatography (hexanes/ethyl acetate = 9:1) to give **2**. White solid, mp: 95.9-96.8 °C (755 mg, 85%); ^{1}**H NMR** (400 MHz, $CDCl_3$) δ 10.18 (s, 1H), 7.07 (s, 1H), 6.67 (s, 1H), 3.91 (s, 3H), 3.86 (s, 3H); ^{13}**C NMR** (100 MHz, $CDCl_3$) δ 196.4, 161.4, 159.1, 136.7, 105.1, 104.8, 84.3, 56.9, 56.0; **HRMS** (ESI-QTOF) *m/z* $[M+H]^+$ calcd for $C_9H_{10}IO_3$ 292.9669 found 292.9667.

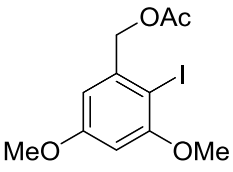

3

2-Iodo-3,5-dimethoxybenzyl acetate (3). The compound **7** (4.5 g, 15.3 mmol) was dissolved in pyridine (7 mL) and Ac_2O (7 mL) at rt. After being stirred for 16 h, the reaction mixture was diluted with ethyl acetate (30 mL) and washed with 1M HCl (20 mL × 3). The combined organic layers were dried over $MgSO_4$ and concentrated in vacuo. The residue was purified by silica gel column chromatography (hexanes/ethyl acetate = 10:1) to give **3**. White solid,

mp: 75.8-76.2 °C (4.9 g, 96%); ^{1}**H NMR** (400 MHz, $CDCl_3$) δ 6.62 (s,1H), 6.41 (s, 1H), 5.14 (s, 2H), 3.87 (s, 3H), 3.83 (s, 3H), 2.15 (s, 3H); ^{13}**C NMR** (100 MHz, $CDCl_3$) δ 170.7, 161.3, 159.1, 140.3, 106.6, 98.4, 79.3, 77.5, 77.2, 70.5, 56.7, 55.7, 21.1; **HRMS** (ESI-QTOF) *m/z* $[M+H]^+$ calcd for $C_{11}H_{14}IO_4$ 336.9931 found 336.9930.

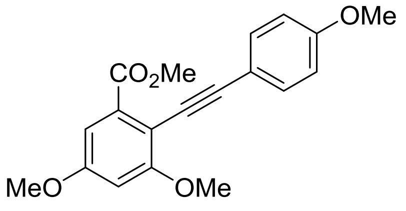

4

Methyl 3,5-dimethoxy-2-((4-methoxyphenyl)ethynyl)benzoate (4). To a stirred solution of **1** (150 mg, 0.47 mmol) in Et_3N (2 mL) were added $(Ph_3P)_2PdCl_2$ (16 mg, 0.05 equiv), CuI (4 mg, 0.05 equiv), and 4-ethynylanisole (68 mg, 1.1 equiv). After being heated at 60 °C for 5 h, the reaction mixture was concentrated under reduced pressure to give the crude residue, which was purified by silica gel column chromatography (hexanes/ethyl acetate = 17:3) to give **4**. Pale brown solid, mp: 98.4-99.2 °C (151 mg, 99%); ^{1}**H NMR** (400 MHz, $CDCl_3$) δ 7.50 (d, *J* = 8.8 Hz, 2H), 7.03 (d, *J* = 1.6 Hz, 1H), 6.87 (d, *J* = 8.4 Hz, 2H), 6.61 (s, 1H), 3.95 (s, 3H), 3.91 (s, 3H), 3.86 (s, 3H), 3.83 (s, 3H); ^{13}**C NMR** (100 MHz, $CDCl_3$) δ 167.2, 161.8, 159.9, 159.6, 134.7, 133.2, 116.3, 114.0, 106.4, 106.0, 102.2, 97.5, 82.6, 56.5, 55.8, 55.4, 52.4; **HRMS** (ESI-QTOF) *m/z* $[M+H]^+$ calcd for $C_{19}H_{19}O_5$ 327.1227 found 327.1225.

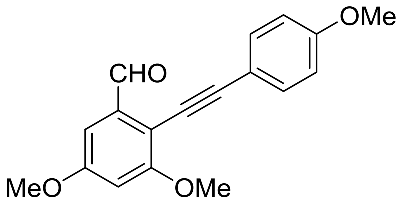

5

3,5-Dimethoxy-2-((4-methoxyphenyl)ethynyl)benzaldehyde (5). To a stirred solution of **2**

(500 mg, 1.71 mmol) in Et_3N (8 mL) were added $(Ph_3P)_2PdCl_2$ (60 mg, 0.05 equiv), CuI (16 mg, 0.05 equiv), and 4-ethynylanisole (249 mg, 1.1 equiv). After being heated at 60 °C for 5 h, the reaction mixture was concentrated under reduced pressure to give the crude residue, which was purified by silica gel column chromatography (hexanes/ethyl acetate = 17:3) to give **5**. Yellow solid, mp: 90.1-90.9 °C (488 mg, 96%); **^{1}H NMR** (400 MHz, $CDCl_3$) δ 10.62 (s, 1H), 7.50 (d, *J* = 8.8 Hz, 2H), 7.03 (d, *J* = 1.6 Hz, 1H), 6.88 (d, *J* = 8.4 Hz, 2H), 6.67 (d, *J* = 1.6 Hz, 1H), 3.92 (s, 3H), 3.87 (s, 3H), 3.83 (s, 3H); **^{13}C NMR** (100 MHz, $CDCl_3$) δ 192.0, 161.6, 160.5, 160.0, 137.8, 133.1, 115.2, 114.2, 110.7, 104.6, 101.1, 99.4, 79.8, 56.4, 55.8, 55.4; **HRMS** (ESI-QTOF) *m/z* $[M+H]^+$ calcd for $C_{18}H_{17}O_4$ 297.1121 found 297.1122.

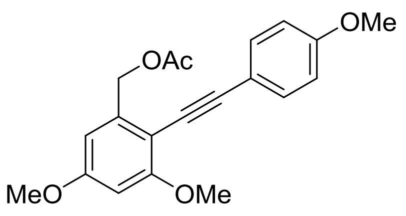

6

3,5-Dimethoxy-2-((4-methoxyphenyl)ethynyl)benzyl acetate (6). To a stirred solution of **3** (500 mg, 1.48 mmol) in Et_3N (7 mL) were added $(Ph_3P)_2PdCl_2$ (52 mg, 0.05 equiv), CuI (14 mg, 0.05 equiv), and 4-ethynylanisole (216 mg, 1.1 equiv). After being heated at 60 °C for 5 h, the reaction mixture was concentrated under reduced pressure to give the crude residue, which was purified by silica gel column chromatography (hexanes/ethyl acetate = 9:1) to give **6**. Pale yellow solid, mp: 92.0-92.6 °C (405 mg, 80%); **^{1}H NMR** (400 MHz, $CDCl_3$) δ 7.47 (d, *J* = 8.8 Hz, 2H), 6.86 (d, *J* = 8.8 Hz, 2H), 6.57 (s, 1H), 6.44 (s, 1H), 5.31 (s, 2H), 3.89 (s, 3H), 3.84 (s, 3H), 3.82 (s, 3H), 2.12 (s, 3H); **^{13}C NMR** (100 MHz, $CDCl_3$) δ 170.9, 161.4, 160.6, 159.6, 140.2, 133.0, 116.0, 114.0, 105.2, 104.9 98.0, 97.4, 81.5, 65.1, 56.2, 55.6, 55.4, 21.1; **HRMS** (ESI-QTOF) *m/z* $[M+H]^+$ calcd for $C_{20}H_{21}O_5$ 341.1384 found 341.1386.

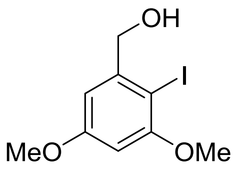

7

(2-Iodo-3,5-dimethoxyphenyl)methanol (7). To a stirred solution of 3,5-dimethoxybenzyl alcohol (2.6 g, 15.5 mmol) in DMF (30 mL) was portionwise added NIS (3.5 g, 1.0 equiv) at 0 °C. After being heated at 40 °C for 3 h, the reaction mixture was diluted with the ethyl acetate (20 mL) and washed with 10% aqueous Na_2SO_3 solution and brine. The combined organic layers were dried over $MgSO_4$ and concentrated in vacuo. The residue was used for the next step without further purification. White solid, mp: 93.8-94.2 °C (4.5 g, 99%); **^{1}H NMR** (400 MHz, $CDCl_3$) δ 6.74 (s, 1H), 6.39 (s, 1H), 4.68 (d, *J* = 5.6 Hz, 2H), 3.87 (s, 3H), 3.83 (s, 3H), 2.14 (t, *J* = 6.0 Hz, 1H); **^{13}C NMR** (100 MHz, $CDCl_3$) δ 161.5, 158.8, 144.9, 105.4, 98.2, 78.0, 69.8, 56.6, 55.7; **HRMS** (ESI-QTOF) *m/z* $[M+H]^+$ calcd for $C_9H_{12}IO_3$ 294.9826 found 294.9826.

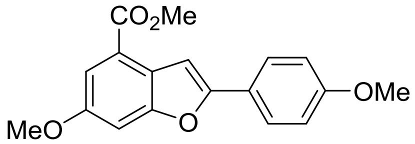

8

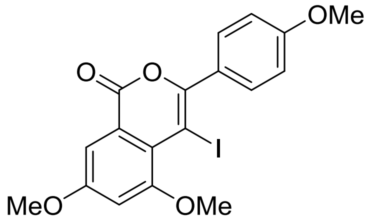

9

Methyl 6-methoxy-2-(4-methoxyphenyl)benzofuran-4-carboxylate (8) and 4-iodo-5,7-dimethoxy-3-(4-methoxyphenyl)-1H-isochromen-1-one (9). To a stirred solution of compound **4** (33 mg, 0.1 mmol) in CH_2Cl_2 (2 mL) were added I_2 (77 mg, 3.0 equiv) and $NaHCO_3$ (26 mg, 3.0 equiv) at 0 °C. After being stirred at rt for 1 h, the reaction mixture was diluted with CH_2Cl_2 (5 mL) and washed with saturated aqueous Na_2SO_3 (5 mL × 3). The

combined organic layers were dried over $MgSO_4$ and concentrated in vacuo. The residue was purified by silica gel column chromatography (hexanes/ethyl acetate/dichloromethane = 20:1:2) to give **8** and **9**. **8:** White solid, mp: 105.4-106.1 °C (10 mg, 32%); **^{1}H NMR** (400 MHz, $CDCl_3$) δ 7.81(d, *J* = 8.4 Hz, 2H), 7.57 (s, 1H), 7.39 (s, 1H), 7.26 (s, 1H), 6.98 (d, *J* = 8.8 Hz, 2H), 4.00 (s, 3H), 3.91 (s, 3H), 3.87 (s, 3H); **^{13}C NMR** (100 MHz, $CDCl_3$) δ 167.0, 160.2, 157.2, 156.9, 156.2, 126.5, 124.2, 123.2, 121.9, 114.5, 112.6, 101.7, 100.7, 56.2, 55.5, 52.2; **HRMS** (ESI-QTOF) *m/z* $[M+H]^+$ calcd for $C_{18}H_{17}O_5$ 313.1071 found 313.1072.

9: Pale yellow solid, mp: decomposed at 157 °C (29 mg, 66%); **^{1}H NMR** (400 MHz, $CDCl_3$) δ 7.59 (d, *J* = 8.4 Hz, 2H), 7.42 (d, *J* = 1.6 Hz, 1H), 6.96 (d, *J* = 8.4 Hz, 2H), 6.86 (d, *J* = 1.6 Hz, 1H), 3.93 (s, 3H), 3.91 (s, 3H), 3.87 (s, 3H); **^{13}C NMR** (100 MHz, $CDCl_3$) δ 162.1, 160.7, 160.4, 156.2, 153.1, 132.1, 129.0, 123.5, 121.1, 113.5, 107.1, 102.5, 61.9, 56.0, 55.6, 55.5; **HRMS** (ESI-QTOF) *m/z* $[M+H]^+$ calcd for $C_{18}H_{16}IO_5$ 439.0037 found 439.0038.

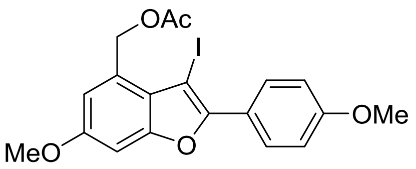

11

(3-Iodo-6-methoxy-2-(4-methoxyphenyl)benzofuran-4-yl)methyl acetate (11). To a stirred solution of **6** (1.0 g, 2.94 mmol) in CH_2Cl_2 (10 mL) were added I_2 (2.2 g, 3.0 equiv) and $NaHCO_3$ (740 mg, 3.0 equiv) at 0 °C. After being stirred at rt for 18 h, the reaction mixture was washed with saturated aqueous Na_2SO_3 (10 mL × 3). The combined organic layer was dried over $MgSO_4$ and concentrated in vacuo. The residue was purified by silica gel column chromatography (hexanes/ethyl acetate/dichloromethane = 50:1:2) to give **11**. White solid, mp: 132.8-133.5 °C (1.12 g, 84%); **^{1}H NMR** (400 MHz, $CDCl_3$) δ 7.98 (d, *J* = 8.4 Hz, 2H), 7.03 (s, 1H), 7.00 (d, *J* = 8.8 Hz, 2H), 6.91 (s, 1H), 5.59 (s, 2H), 3.872 (s, 3H), 3.865 (s, 3H),

2.16 (s, 3H); **^{13}C NMR** (100 MHz, $CDCl_3$) δ 170.9, 160.3, 157.8, 155.7, 153.9, 129.7, 129.2, 122.8, 122.0, 114.2 114.0, 96.1, 62.4, 55.9, 55.7, 55.5, 21.5; **HRMS** (ESI-QTOF) *m/z* $[M+H]^+$ calcd for $C_{19}H_{18}IO_5$ 453.0193 found 453.0192.

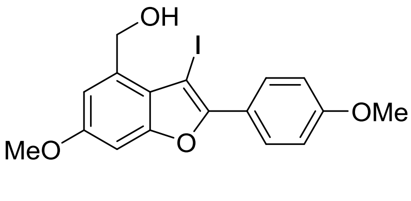

12

(3-Iodo-6-methoxy-2-(4-methoxyphenyl)benzofuran-4-yl)methanol (12). To a stirred solution of compound **11** (1.0 g, 2.2 mmol) in MeOH (7 mL) was added K_2CO_3 (917 mg, 3.0 equiv). After being stirred at rt for 2 h, the reaction mixture was concentrated under reduced pressure and extracted with CH_2Cl_2 (10 mL). The aqueous layer was extracted with CH_2Cl_2 (5 mL × 2) two more times. The combined organic layers were dried over $MgSO_4$ and concentrated in vacuo. The residue was used for the next step without further purification. Pale yellow solid, mp: 141.8-142.4 °C (906 mg, 100%); **^{1}H NMR** (400 MHz, $CDCl_3$) δ 7.97 (d, J = 8.8 Hz, 2H), 7.05-6.93 (m, 4H), 5.21 (s, 2H), 3.873 (s, 3H), 3.865 (s, 3H); **^{13}C NMR** (100 MHz, $CDCl_3$) δ 160.3, 158.1, 155.8, 153.6, 134.6, 129.6, 122.8, 121.3, 114.0, 112.6, 95.5, 61.0, 55.9, 55.5; **HRMS** (ESI-QTOF) *m/z* $[M+H]^+$ calcd for $C_{17}H_{16}IO_4$ 411.0088 found 411.0086.

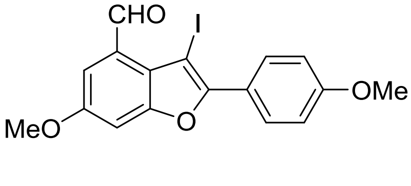

13

3-Iodo-6-methoxy-2-(4-methoxyphenyl)benzofuran-4-carbaldehyde (13). To a stirred

solution of **12** (906 mg, 2.2 mmol) in dry CH_2Cl_2 (10 mL) was added Dess–Martin periodinane (1124 mg, 1.2 equiv) at 0 °C. After being stirred at rt for 2 h, the reaction mixture was suction-filtered through a pad of Celite and the filtrate was concentrated under reduced pressure to give the residue which was purified by silica gel column chromatography (hexanes/ethyl acetate/dichloromethane = 20:1:2) to give compound **13**. Yellow solid, mp: 159.2-159.9 °C (900 mg, 100%); ^{1}**H NMR** (400 MHz, $CDCl_3$) δ 11.59 (s, 1H), 8.01 (d, *J* = 8.8 Hz, 2H), 7.54 (d, *J* = 1.2 Hz, 1H), 7.26 (s, 1H), 7.03 (d, *J* = 8.8 Hz, 2H), 3.91 (s, 3H), 3.89 (s, 3H); ^{13}**C NMR** (100 MHz, $CDCl_3$) δ 187.7, 160.8, 157.5, 156.6, 156.3, 130.2, 129.4, 126.1, 122.1, 114.1, 109.2, 103.0, 56.2, 55.6, 53.1; **HRMS** (ESI-QTOF) *m/z* $[M+H]^+$ calcd for $C_{17}H_{14}IO_4$ 408.9931 found 408.9930.

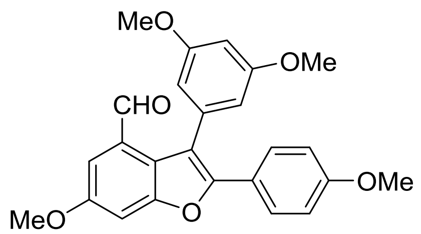

14

3-(3,5-Dimethoxyphenyl)-6-methoxy-2-(4-methoxyphenyl)benzofuran-4-carbaldehyde (14). Similar as already described in [S1] a mixture of **13** (850 mg, 2.08 mmol), $Pd(Ph_3P)_4$ (241 mg, 0.1 equiv), K_2CO_3 (576 mg, 2.0 equiv), and 3,5-dimethoxyphenylboronic acid (758 mg, 2.0 equiv) in toluene/EtOH/H_2O (4:2:1, 14 mL) was heated at 100 °C for 3 h. The reaction mixture was concentrated under reduced pressure, diluted with CH_2Cl_2 (15 mL), and washed with H_2O (15 mL). The aqueous layer was extracted with CH_2Cl_2 (15 mL × 2) two more times. The combined organic layers were dried over $MgSO_4$ and concentrated in vacuo. The residue was purified by silica gel column chromatography (hexanes/ethyl acetate/dichloromethane = 50:1:2) to give **14**. Yellow solid, mp: 162.1-162.7 °C (767 mg, 88%); ^{1}**H NMR** (400 MHz, $CDCl_3$) δ 9.78 (s, 1H), 7.53 (d, *J* = 8.8 Hz, 2H), 7.46 (d, *J* = 2.0

Hz, 1H), 7.32 (d, J = 2.0 Hz, 1H), 6.83 (d, J = 8.8 Hz, 2H), 6.61 (d, J = 1.6 Hz, 2H), 6.56 (s, 1H), 3.92 (s, 3H), 3.80 (s, 3H), 3.79 (s, 6H); **^{13}C NMR** (100 MHz, $CDCl_3$) δ 189.7, 161.9, 159.8, 157.4, 155.2, 152.2, 136.3, 129.3, 127.9, 126.7, 122.6, 114.9, 114.0, 107.8, 107.4, 103.1, 100.6, 56.1, 55.4, 55.3; **HRMS** (ESI-QTOF) *m/z* $[M+H]^+$ calcd for $C_{25}H_{23}O_6$ 419.1489 found 419.1488.

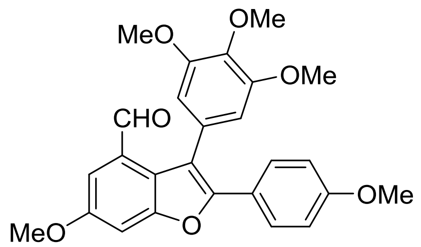

15

6-Methoxy-2-(4-methoxyphenyl)-3-(3,4,5-trimethoxyphenyl)benzofuran-4-carbaldehyde (15). Similar as already described in [S1] a mixture of **13** (60 mg, 0.15 mmol), $Pd(Ph_3P)_4$ (17 mg, 0.1 equiv), K_2CO_3 (41 mg, 2.0 equiv), and 3,4,5-trimethoxyphenylboronic acid (62 mg, 2.0 equiv) in toluene/EtOH/H_2O (4:2:1, 1.75 mL) was heated at 100 °C for 3 h. The reaction mixture was concentrated under reduced pressure, diluted with CH_2Cl_2 (5 mL), and washed with H_2O (5 mL). The aqueous layer was extracted with CH_2Cl_2 (5 mL × 2) two more times. The combined organic layers were dried over $MgSO_4$ and concentrated in vacuo. The residue was purified by silica gel column chromatography (hexanes/ethyl acetate/dichloromethane = 20:1:2) to give **15**. Yellow solid, mp: 171.4-172.2 °C (59 mg, 89%); **^{1}H NMR** (400 MHz, $CDCl_3$) δ 9.81 (s, 1H), 7.50 (d, J = 8.4 Hz, 2H), 7.47 (s, 1H), 7.34 (s, 1H), 6.84 (d, J = 8.8 Hz, 2H), 6.68 (s, 2H), 3.97 (s, 3H), 3.92 (s, 3H), 3.81 (s, 9H); **^{13}C NMR** (100 MHz, $CDCl_3$) δ 189.8, 160.0, 157.6, 155.4, 154.5, 152.5, 138.2, 129.9, 129.5, 128.0, 126.9, 122.6, 115.0, 114.2, 107.6, 106.8, 103.3, 61.3, 56.4, 56.3, 55.4; **HRMS** (ESI-QTOF) *m/z* $[M+H]^+$ calcd for $C_{26}H_{25}O_7$ 449.1595 found 449.1596.

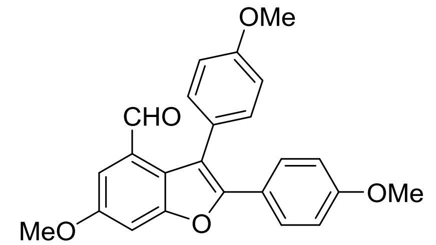

16

6-Methoxy-2,3-bis(4-methoxyphenyl)benzofuran-4-carbaldehyde (16). Similar as already described in [S1] a mixture of **13** (60 mg, 0.15 mmol), $Pd(Ph_3P)_4$ (17 mg, 0.1 equiv), K_2CO_3 (41 mg, 2.0 equiv), and 4-methoxyphenylboronic acid (45 mg, 2.0 equiv) in toluene/EtOH/H_2O (4:2:1, 1.75 mL) was heated at 100 °C for 3 h. The reaction mixture was concentrated under reduced pressure, diluted with CH_2Cl_2 (5 mL), and washed with H_2O (5 mL). The aqueous layer was extracted with CH_2Cl_2 (5 mL × 2) two more times. The combined organic layers were dried over $MgSO_4$ and concentrated in vacuo. The residue was purified by silica gel column chromatography (hexanes/ethyl acetate/dichloromethane = 20:1:2) to give **16**. Orange solid, mp: 148.5-149.3 °C (53 mg, 92%); **^{1}H NMR** (400 MHz, $CDCl_3$) δ 9.77 (s, 1H), 7.50-7.41 (m, 3H), 7.35 (d, *J* = 8.4 Hz, 2H), 7.30 (d, *J* = 1.6 Hz, 1H), 7.02 (d, *J* = 8.4 Hz, 2H), 6.80 (d, *J* = 8.8 Hz, 2H), 3.90 (s, 3H), 3.88 (s, 3H), 3.78 (s, 3H); **^{13}C NMR** (100 MHz, $CDCl_3$) δ 189.6, 159.9, 159.8, 157.4, 155.4, 152.7, 131.4, 129.6, 128.1, 127.2, 126.4, 122.9, 115.3, 114.8, 114.1, 107.6, 103.2, 56.2, 55.4, 55.4; **HRMS** (ESI-QTOF) *m/z* $[M+H]^+$ calcd for $C_{24}H_{21}O_5$ 389.1384 found 389.1385.

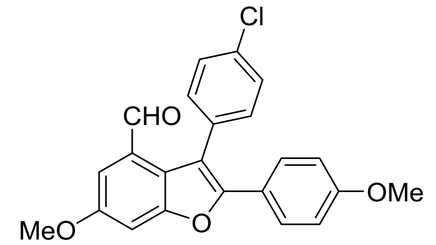

17

3-(4-Chlorophenyl)-6-methoxy-2-(4-methoxyphenyl)benzofuran-4-carbaldehyde (17). A

Similar as already described in [S1] a mixture of **13** (60 mg, 0.15 mmol), $Pd(Ph_3P)_4$ (17 mg, 0.1 equiv), K_2CO_3 (41 mg, 2.0 equiv), and 4-chlorophenylboronic acid (46 mg, 2.0 equiv) in toluene/EtOH/H_2O (4:2:1, 1.75 mL) was heated at 100 °C for 3 h. The reaction mixture was concentrated under reduced pressure, diluted with CH_2Cl_2 (5 mL), and washed with H_2O (5 mL). The water layer was extracted with CH_2Cl_2 (5 mL × 2) two more times. The combined organic layers were dried over $MgSO_4$ and concentrated in vacuo. The residue was purified by silica gel column chromatography (hexanes/ethyl acetate/dichloromethane = 20:1:2) to give **17**. Yellow solid, mp: 128.9-129.4 °C (45 mg, 78%); **^{1}H NMR** (400 MHz, $CDCl_3$) δ 9.76 (s, 1H), 7.49 (d, *J* = 8.4 Hz, 2H), 7.47-7.37 (m, 5H), 7.34 (d, *J* = 2.0 Hz, 1H), 6.83 (d, *J* = 8.8 Hz, 2H), 3.92 (s, 3H), 3.81 (s, 3H); **^{13}C NMR** (100 MHz, $CDCl_3$) δ 189.1, 160.1, 157.6, 155.5, 152.9, 134.8, 133.2, 131.8, 130.2, 129.5, 128.2, 126.3, 122.5, 114.2, 114.0, 108.3, 103.3, 56.2, 55.4; **HRMS** (ESI-QTOF) *m/z* $[M+H]^+$ calcd for $C_{23}H_{18}ClO_4$ 393.0888 found 393.0889.

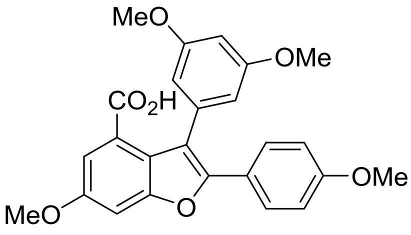

18

3-(3,5-Dimethoxyphenyl)-6-methoxy-2-(4-methoxyphenyl)benzofuran-4-carboxylic acid (18). To a stirred solution of **14** (30 mg, 0.07 mmol) in THF (1.5 mL) were added $NaClO_2$ (65 mg, 10.0 equiv), NaH_2PO_4 (99 mg, 10.0 equiv) in H_2O (0.6 mL), and 2-methyl-2-butene (0.3 mL) at rt. After being stirred at 40 °C for 1 h, the reaction mixture was concentrated under reduced pressure and diluted with CH_2Cl_2 (3 mL) and H_2O (3 mL). The water layer was extracted with CH_2Cl_2 (3 mL × 2) more two times. The combined organic layers were dried over $MgSO_4$ and concentrated in vacuo to give **18**. White solid, mp: decomposed at 112 °C

(31 mg, 100%); **^{1}H NMR** (400 MHz, DMSO-d_6) δ 7.45 (d, *J* = 2.0 Hz, 1H), 7.42 (d, *J* = 8.8 Hz, 2H), 7.09 (d, *J* = 1.6 Hz, 1H), 6.93 (d, *J* = 8.8 Hz, 2H), 6.50 (s, 1H), 6.43 (d, *J* = 1.6 Hz, 2H), 3.86 (s, 3H), 3.75 (s, 3H), 3.70 (s, 6H); **^{13}C NMR** (100 MHz, DMSO-d_6) δ 167.4, 160.3, 159.4, 156.8, 154.7, 150.5, 135.4, 127.8, 127.1, 122.3, 120.3, 115.9, 114.1, 112.0, 107.8, 99.6, 98.6, 56.0, 55.2, 55.1; **HRMS** (ESI-QTOF) *m/z* $[M+H]^+$ calcd for $C_{25}H_{23}O_7$ 435.1438 found 435.1439.

Reference

S1 Jung, Y.; Kim, I. *Asian J. Org. Chem.* **2016,** *5,* 147. doi:10.1002/ajoc.201500423

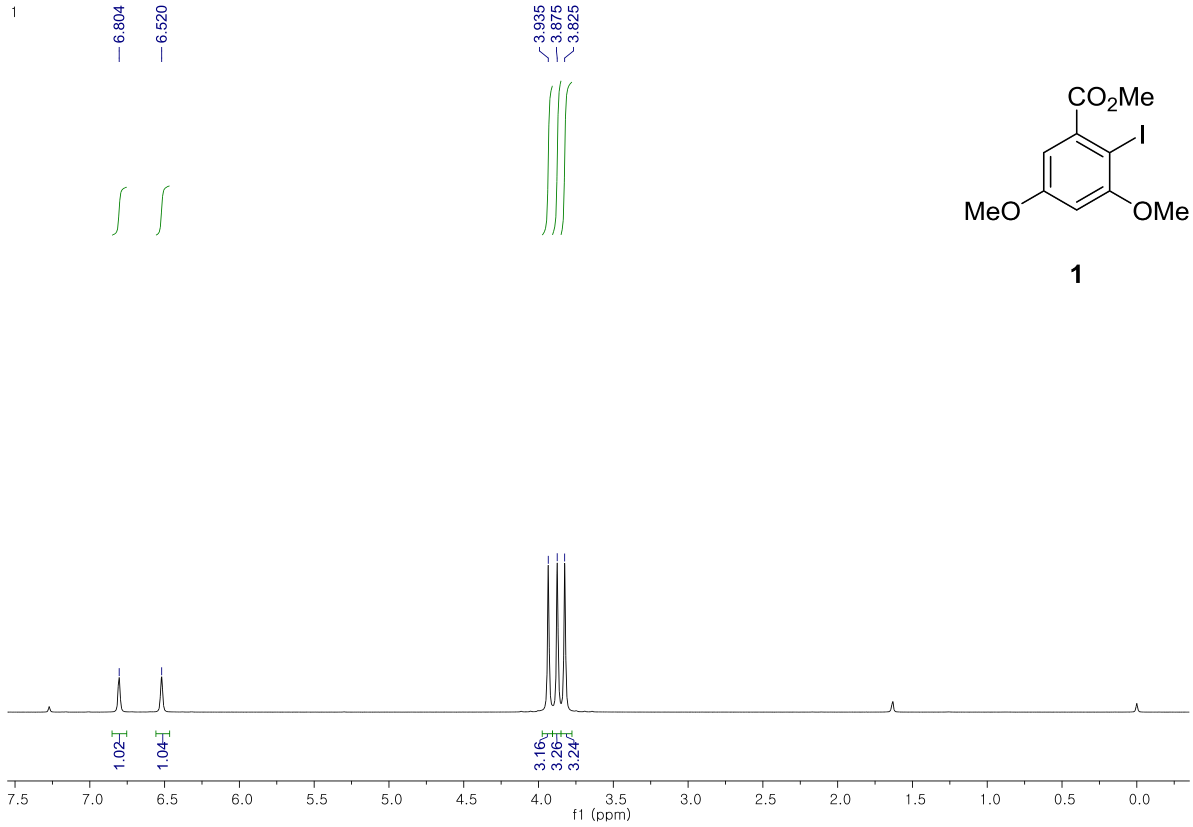
1
6.804
6.520
3.935
3.875
3.825
CO2Me
I
MeO
OMe
1
1.02
1.04
3.16
3.26
3.24
7.5 7.0 6.5 6.0 5.5 5.0 4.5 4.0 3.5 3.0 2.5 2.0 1.5 1.0 0.5 0.0
f1 (ppm)

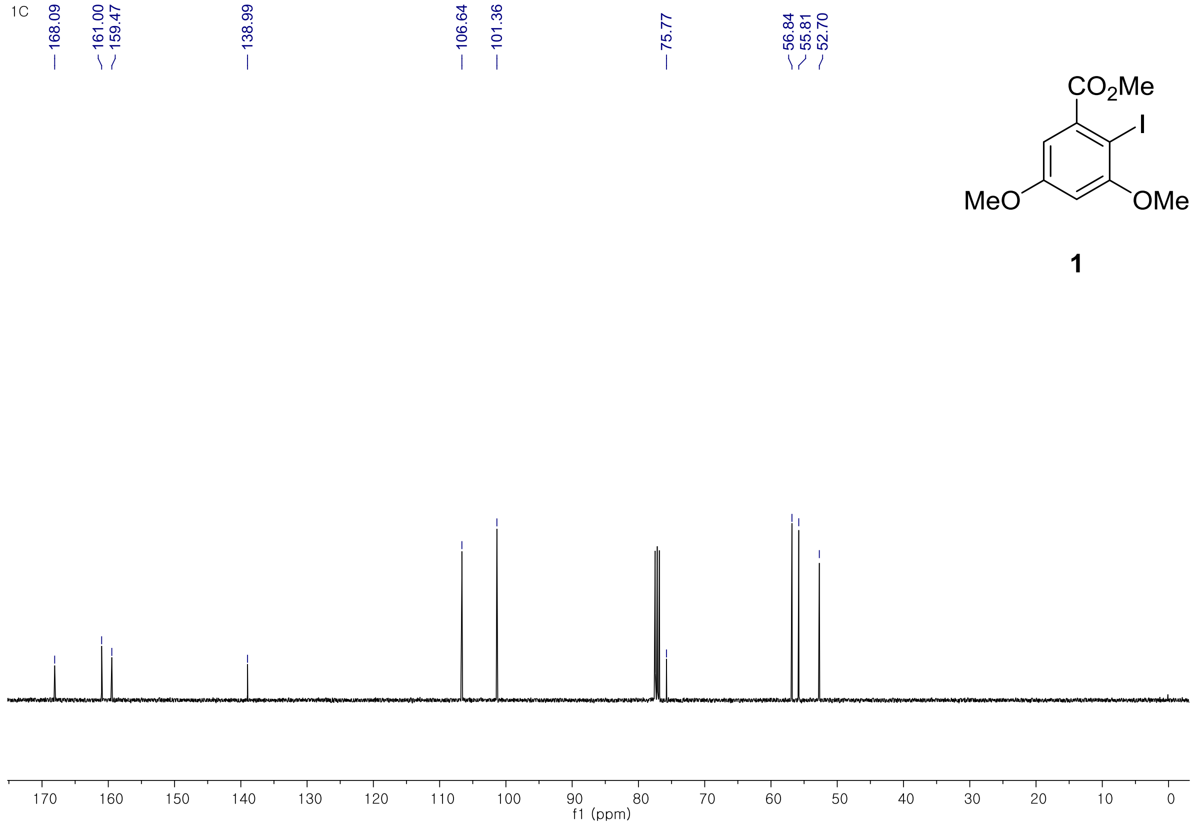
1C
168.09
161.00
159.47
138.99
106.64
101.36
75.77
56.84
55.81
52.70
CO2Me
I
MeO
OMe
1
170 160 150 140 130 120 110 100 90 80 70 60 50 40 30 20 10 0
f1 (ppm)

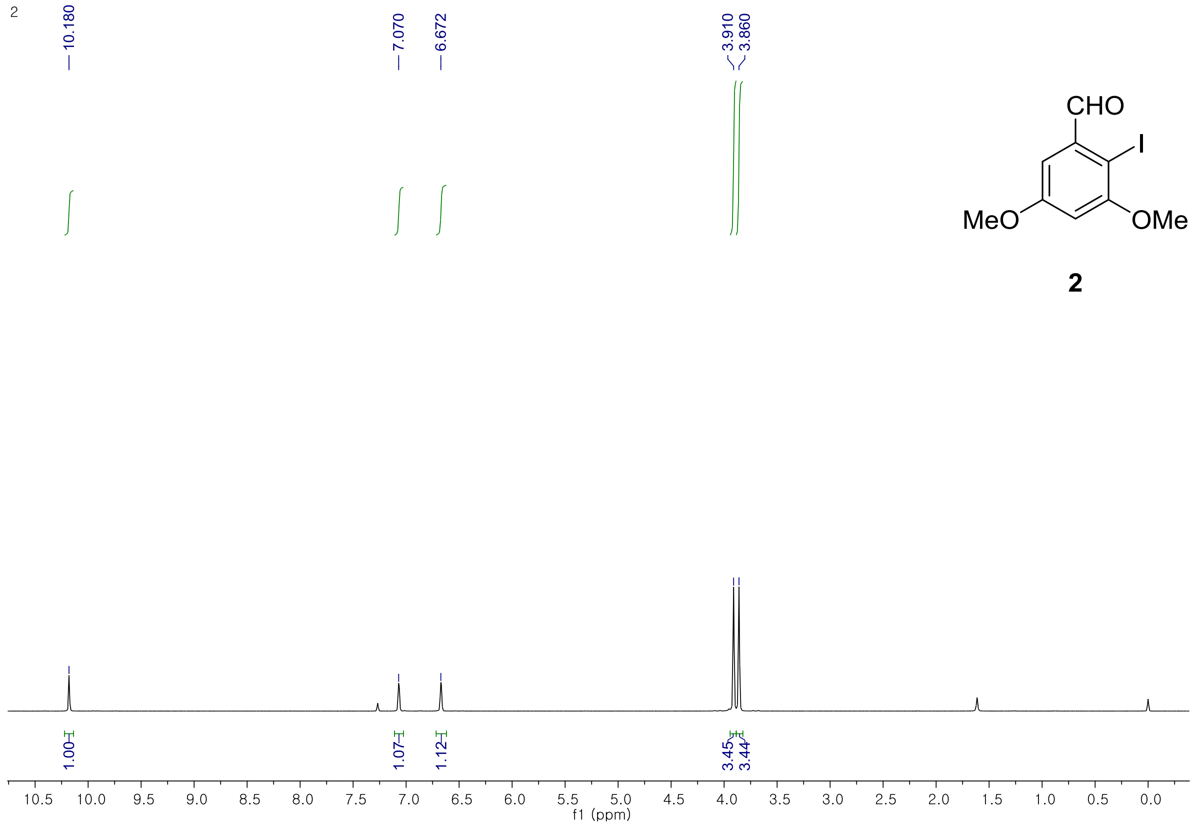
2
10.180
7.070
6.672
3.910
3.860
CHO
I
MeO
OMe
2
1.00
1.07
1.12
3.45
3.44
f1 (ppm)

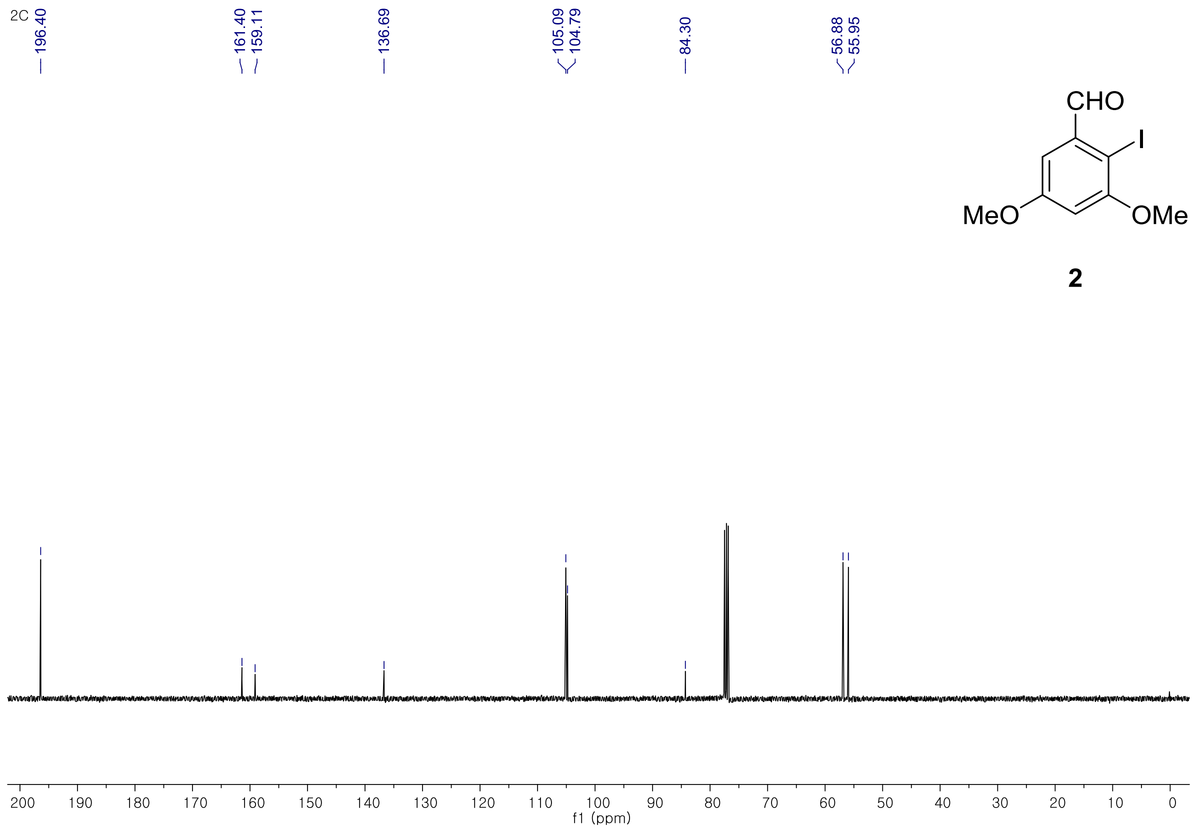
2C
196.40
161.40
159.11
136.69
105.09
104.79
84.30
56.88
55.95
CHO
I
MeO
OMe
2
f1 (ppm)

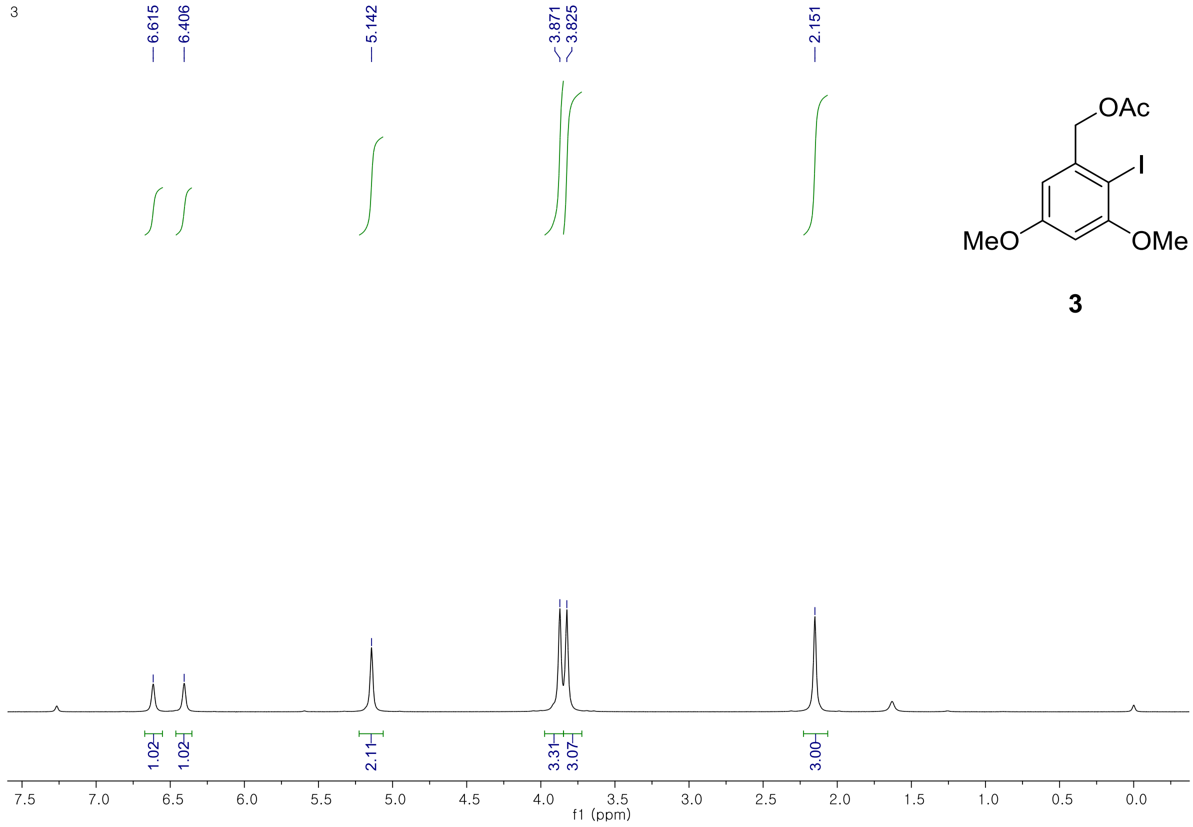
3
6.615
6.406
5.142
3.871
3.825
2.151
OAc
I
MeO
OMe
3
1.02
1.02
2.11
3.31
3.07
3.00
7.5 7.0 6.5 6.0 5.5 5.0 4.5 4.0 3.5 3.0 2.5 2.0 1.5 1.0 0.5 0.0
f1 (ppm)

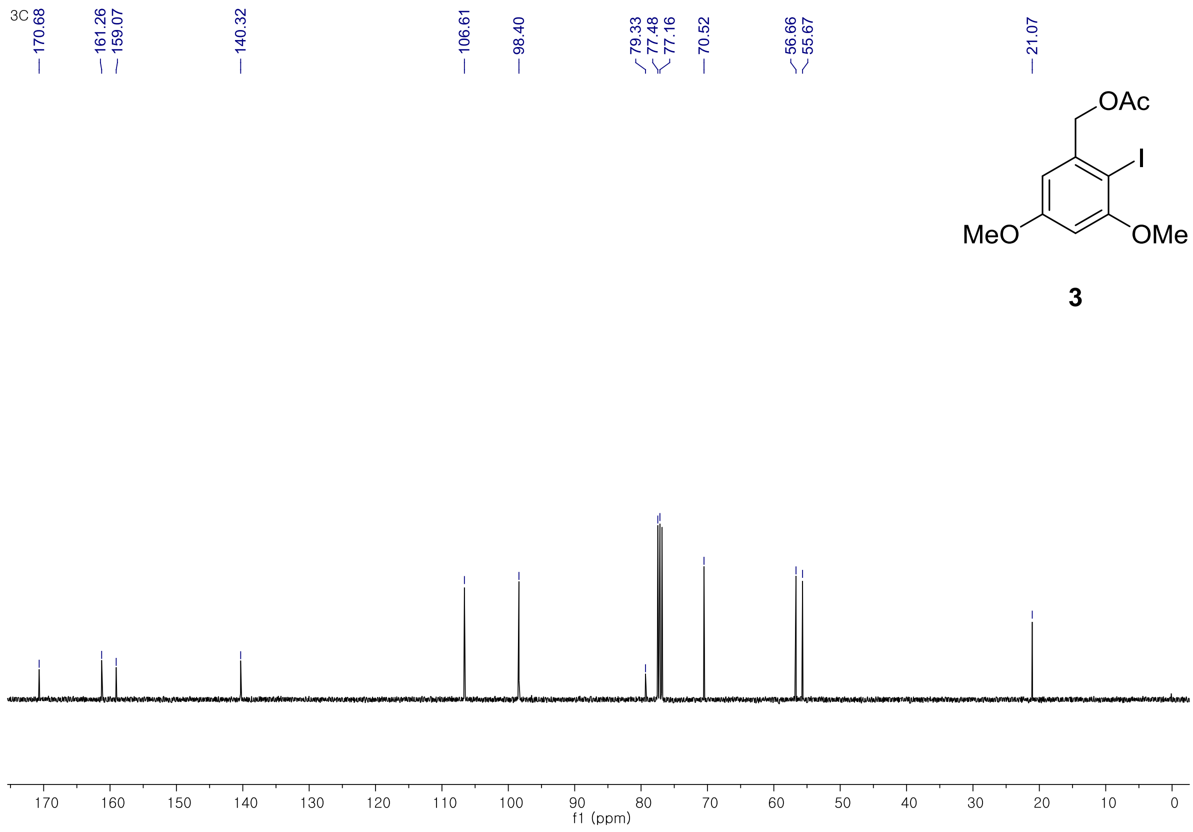
3C
170.68
161.26
159.07
140.32
106.61
98.40
79.33
77.48
77.16
70.52
56.66
55.67
21.07
OAc
I
MeO
OMe
3
170 160 150 140 130 120 110 100 90 80 70 60 50 40 30 20 10 0
f1 (ppm)

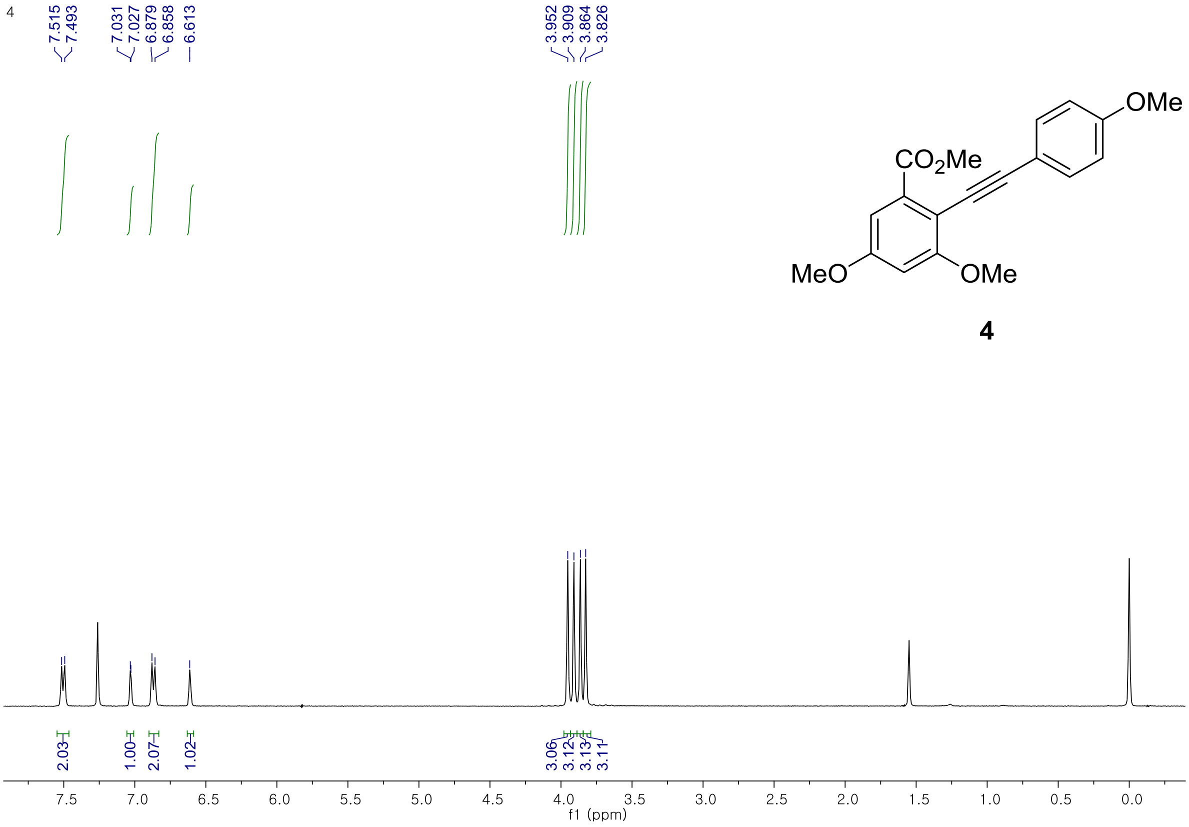
4
7.515
7.493
7.031
7.027
6.879
6.858
6.613
3.952
3.909
3.864
3.826
CO2Me
OMe
MeO
OMe
4
2.03
1.00
2.07
1.02
3.06
3.12
3.13
3.11
f1 (ppm)

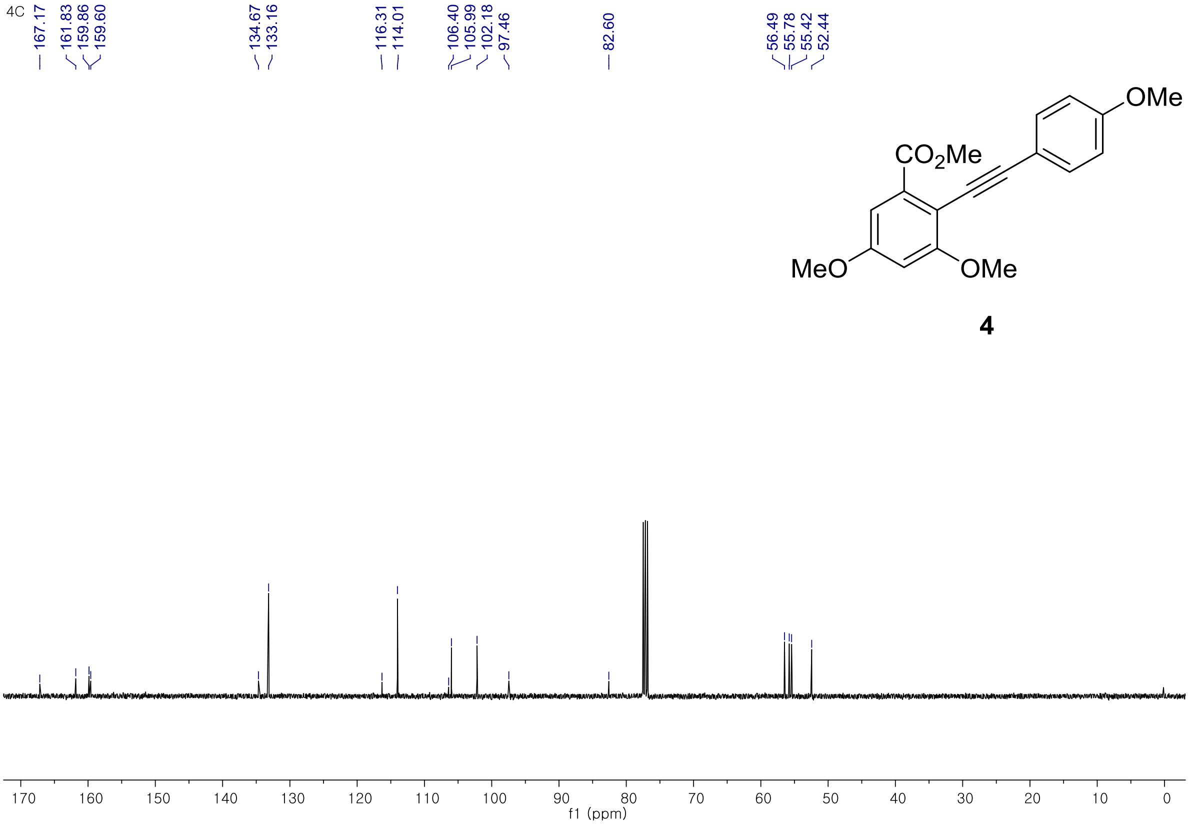
4C
167.17
161.83
159.86
159.60
134.67
133.16
116.31
114.01
106.40
105.99
102.18
97.46
82.60
56.49
55.78
55.42
52.44
CO2Me
OMe
MeO
OMe
4
f1 (ppm)

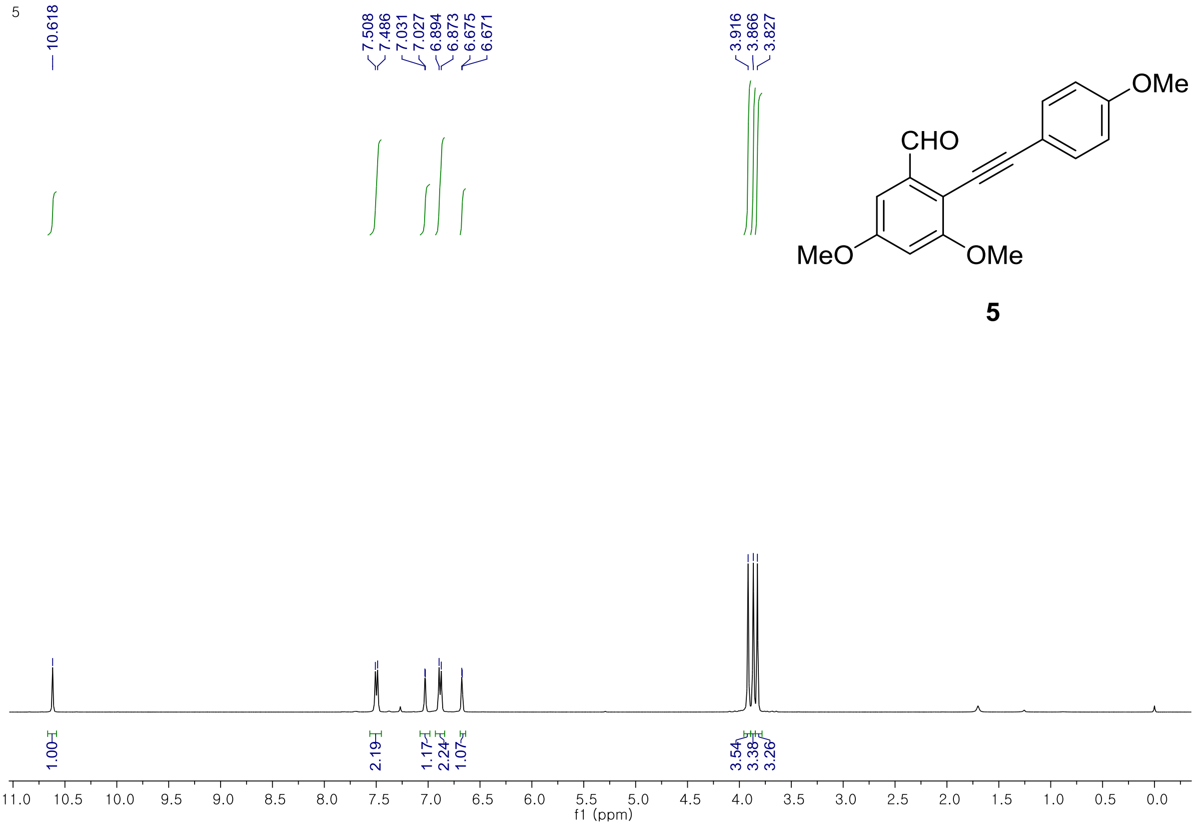
5
10.618
7.508
7.486
7.031
7.027
6.894
6.873
6.675
6.671
3.916
3.866
3.827
CHO
OMe
MeO
OMe
5
1.00
2.19
1.17
2.24
1.07
3.54
3.38
3.26
11.0 10.5 10.0 9.5 9.0 8.5 8.0 7.5 7.0 6.5 6.0 5.5 5.0 4.5 4.0 3.5 3.0 2.5 2.0 1.5 1.0 0.5 0.0
f1 (ppm)

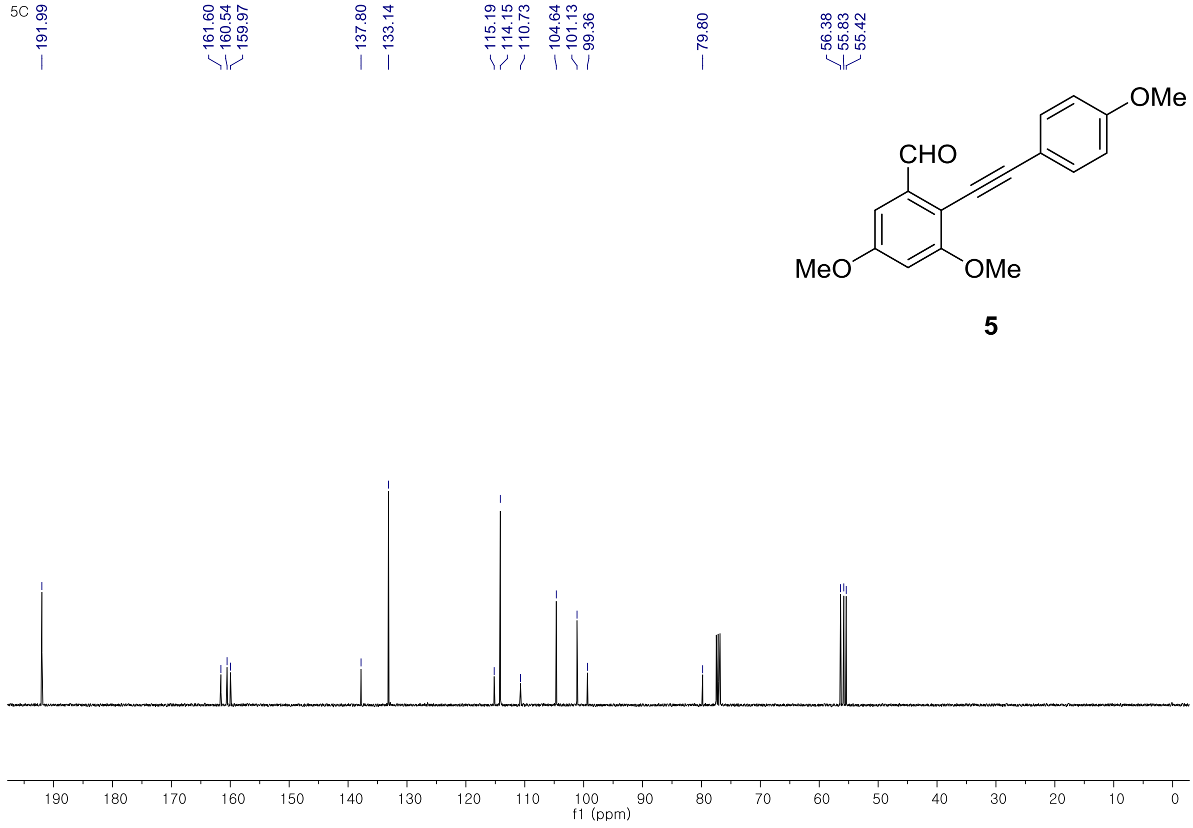
5C
191.99
161.60
160.54
159.97
137.80
133.14
115.19
114.15
110.73
104.64
101.13
99.36
79.80
56.38
55.83
55.42
CHO
OMe
MeO
OMe
5
190 180 170 160 150 140 130 120 110 100 90 80 70 60 50 40 30 20 10 0
f1 (ppm)

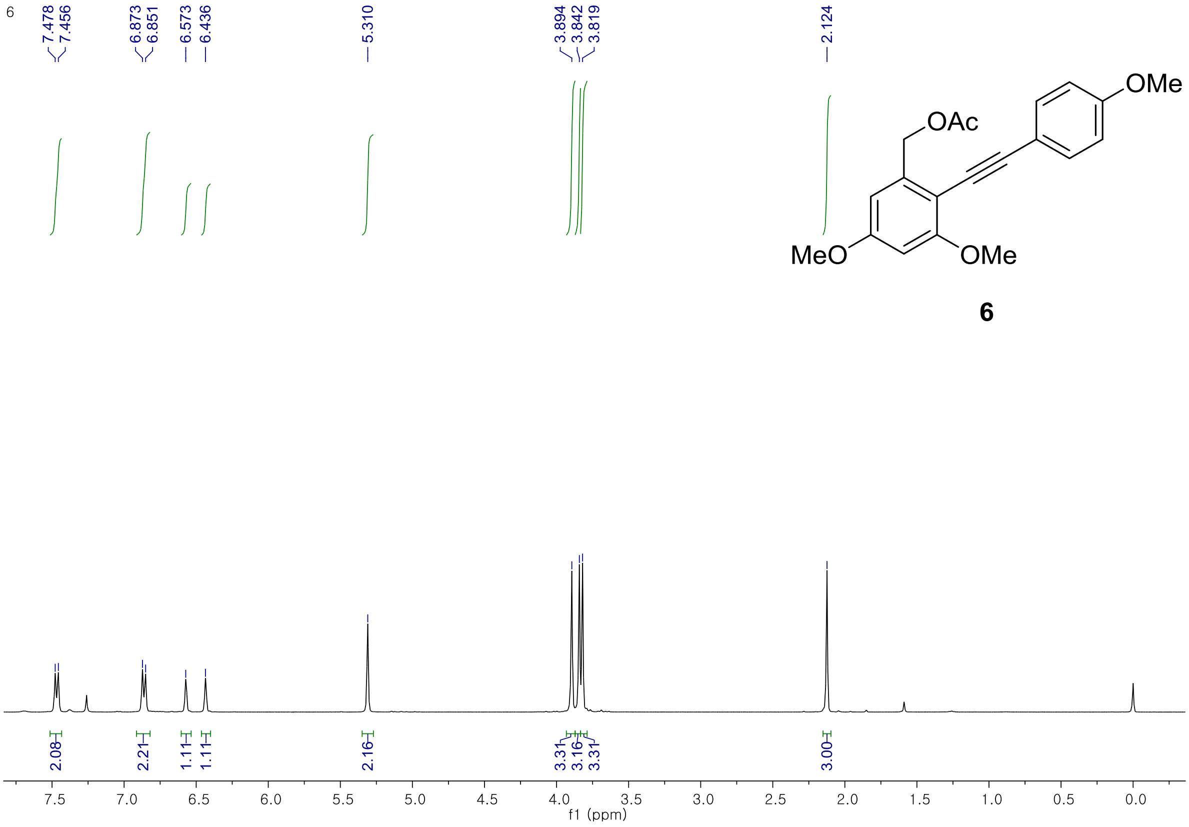
6
7.478
7.456
6.873
6.851
6.573
6.436
5.310
3.894
3.842
3.819
2.124
OAc
OMe
MeO
OMe
6
2.08
2.21
1.11
1.11
2.16
3.31
3.16
3.31
3.00
f1 (ppm)

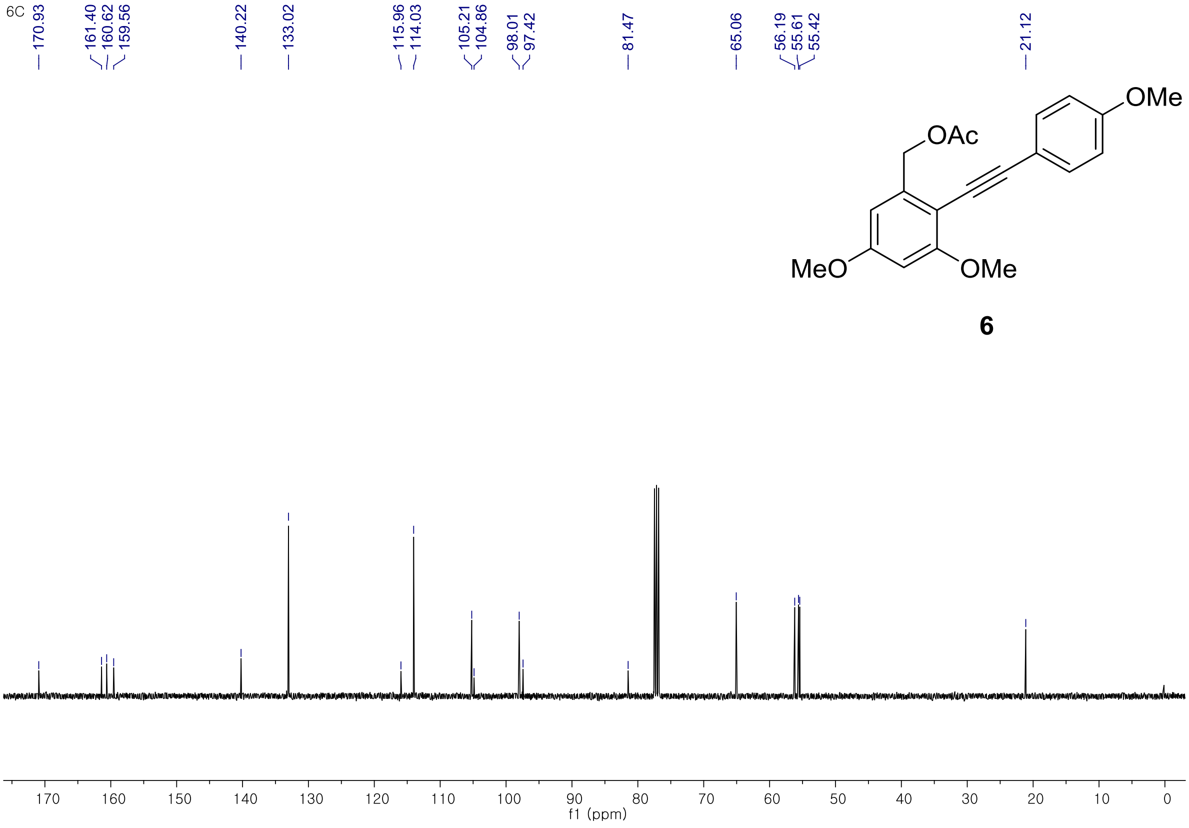
6C
170.93
161.40
160.62
159.56
140.22
133.02
115.96
114.03
105.21
104.86
98.01
97.42
81.47
65.06
56.19
55.61
55.42
21.12
OAc
OMe
MeO
OMe
6
f1 (ppm)

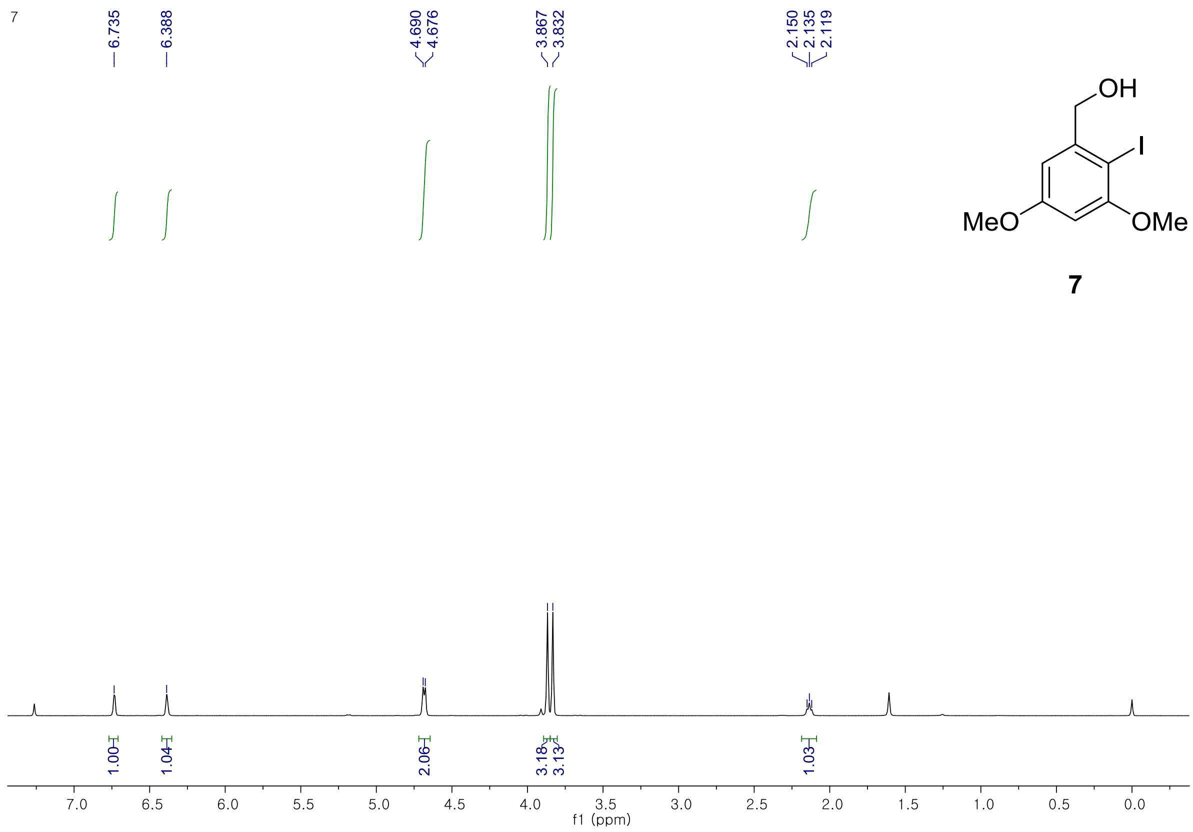
7
6.735
6.388
4.690
4.676
3.867
3.832
2.150
2.135
2.119
OH
I
MeO
OMe
7
1.00
1.04
2.06
3.18
3.13
1.03
7.0 6.5 6.0 5.5 5.0 4.5 4.0 3.5 3.0 2.5 2.0 1.5 1.0 0.5 0.0
f1 (ppm)

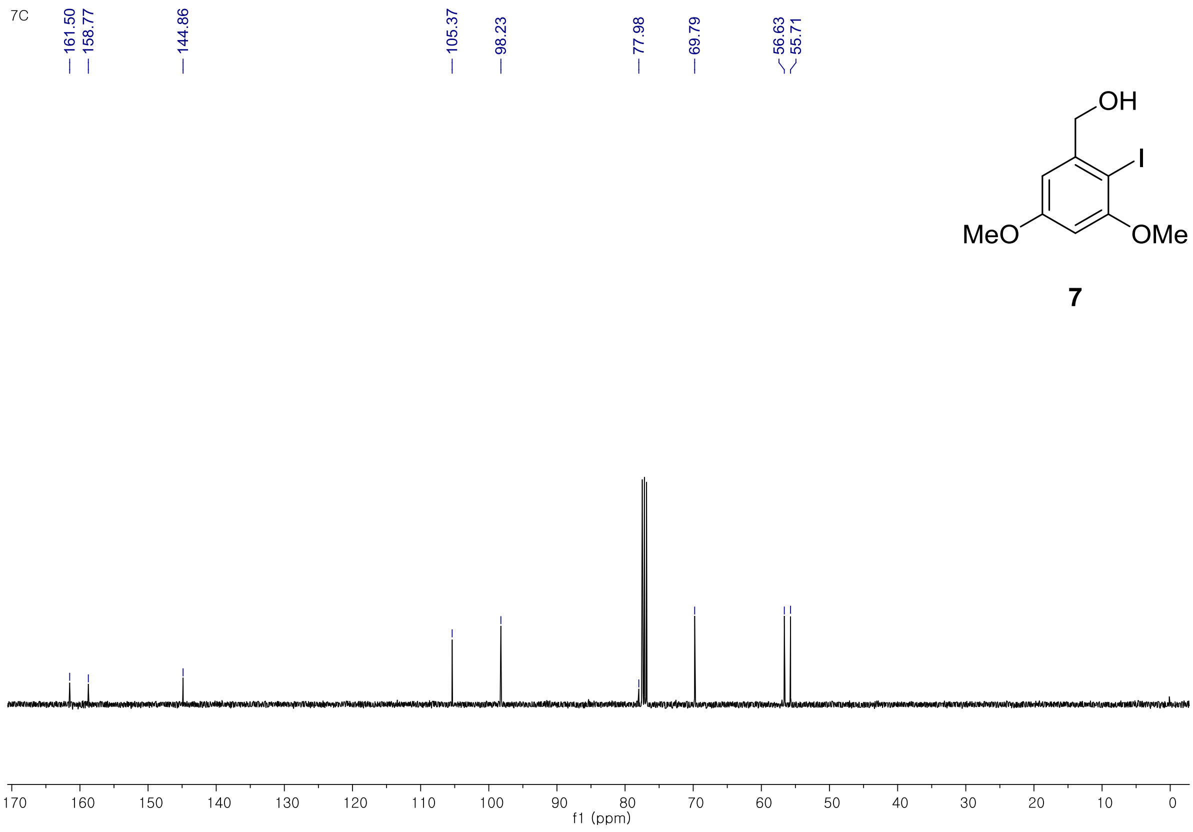
7C
161.50
158.77
144.86
105.37
98.23
77.98
69.79
56.63
55.71
OH
I
MeO
OMe
7
170 160 150 140 130 120 110 100 90 80 70 60 50 40 30 20 10 0
f1 (ppm)

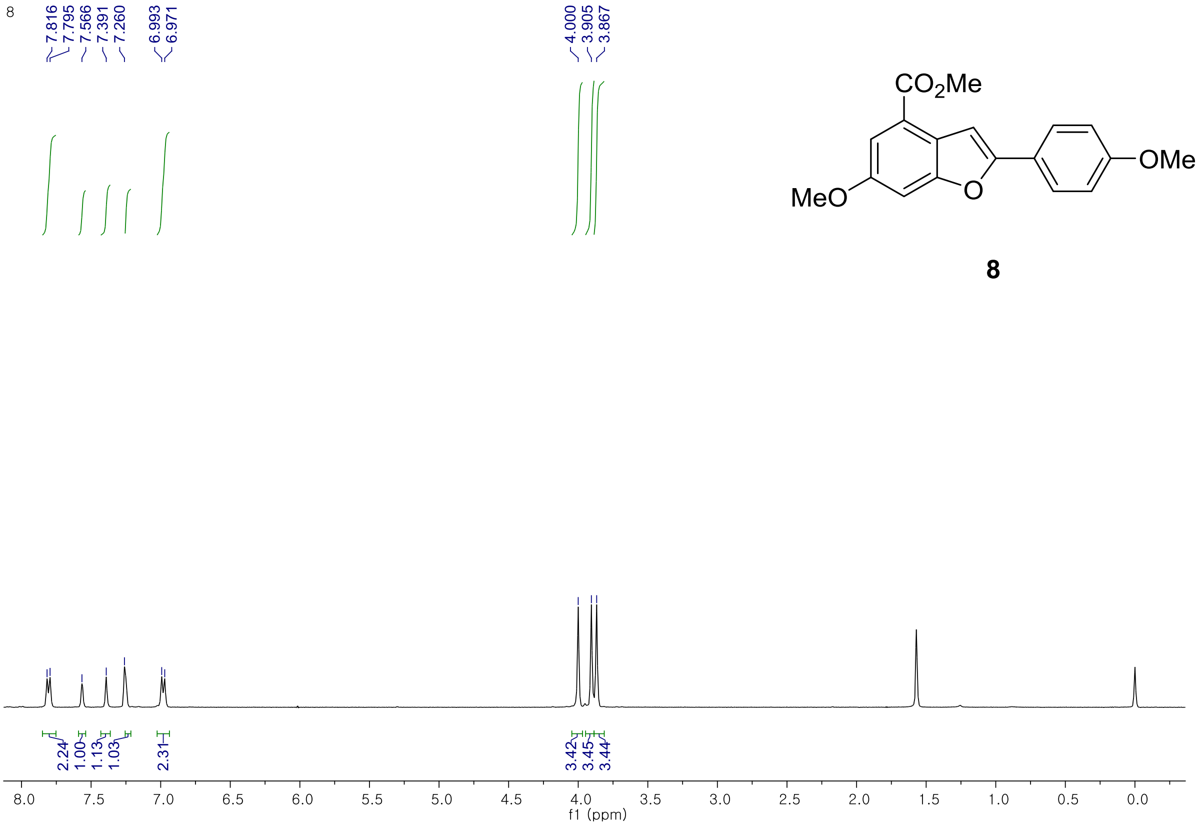
8
7.816
7.795
7.566
7.391
7.260
6.993
6.971
4.000
3.905
3.867
2.24
1.00
1.13
1.03
2.31
3.42
3.45
3.44
f1 (ppm)
CO2Me
MeO
O
OMe

8

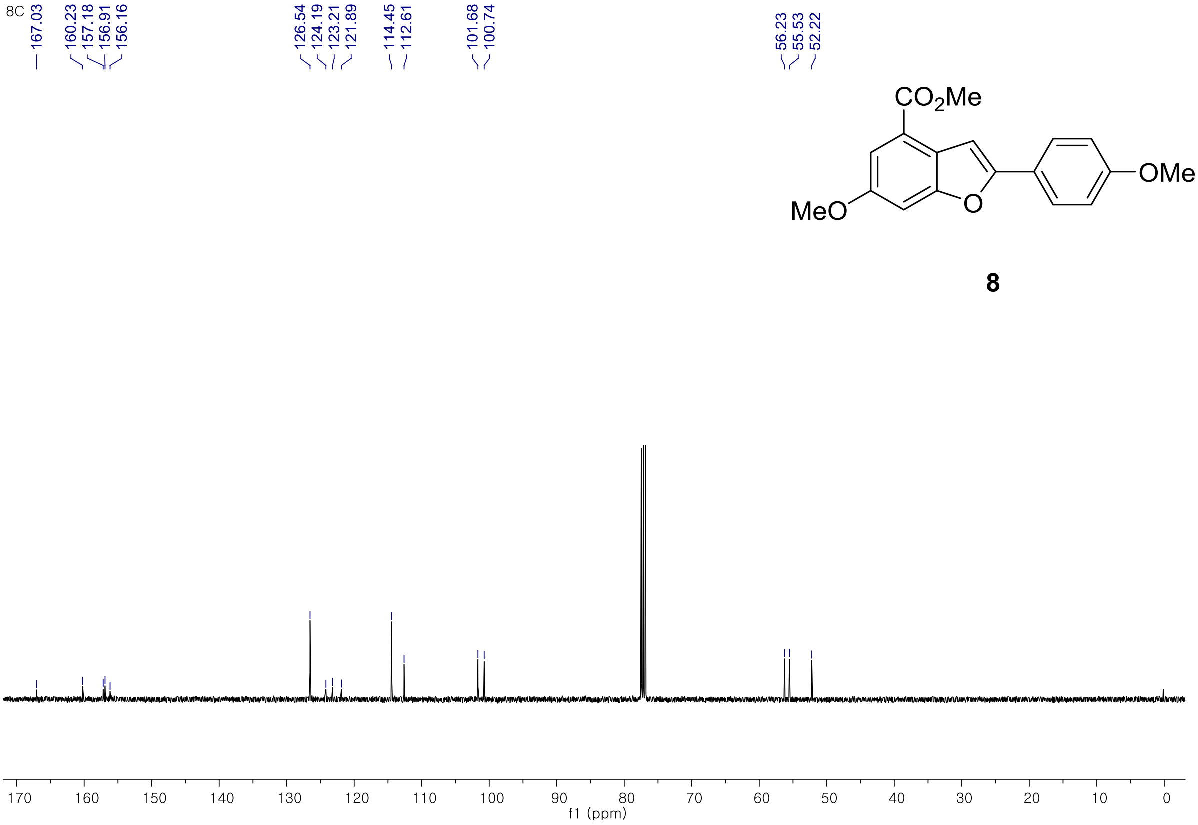
8C
167.03
160.23
157.18
156.91
156.16
126.54
124.19
123.21
121.89
114.45
112.61
101.68
100.74
56.23
55.53
52.22
f1 (ppm)
CO2Me
MeO
O
OMe

8

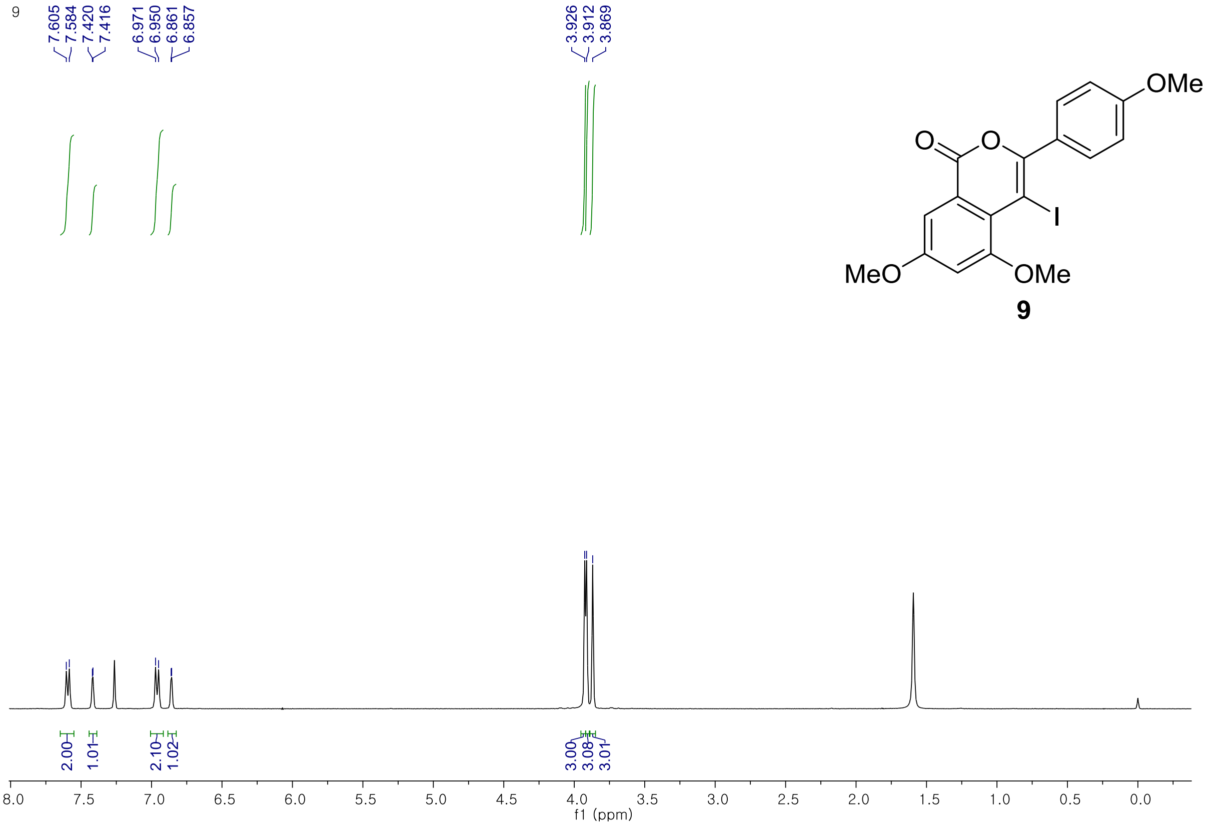
9
7.605
7.584
7.420
7.416
6.971
6.950
6.861
6.857
3.926
3.912
3.869
OMe
O
O
I
MeO
OMe
9
2.00
1.01
2.10
1.02
3.00
3.08
3.01
8.0 7.5 7.0 6.5 6.0 5.5 5.0 4.5 4.0 3.5 3.0 2.5 2.0 1.5 1.0 0.5 0.0
f1 (ppm)

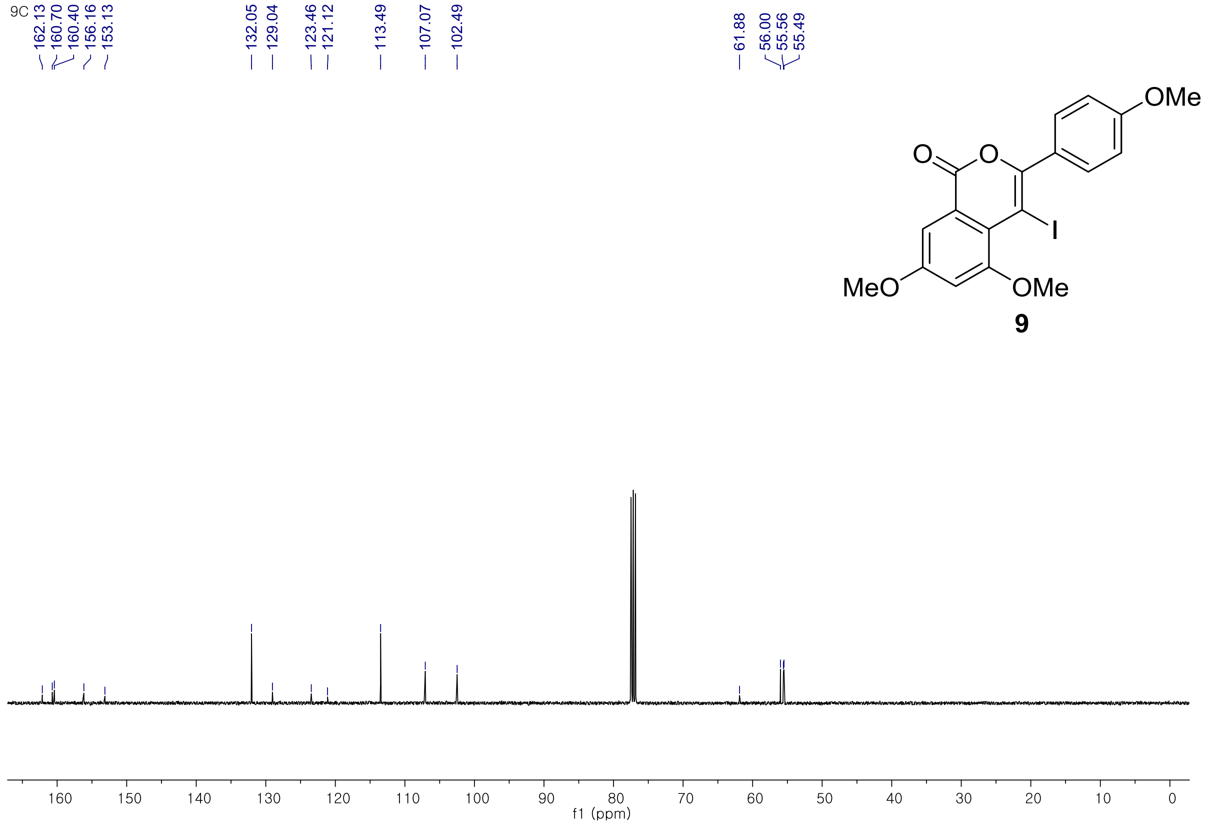
9C
162.13
160.70
160.40
156.16
153.13
132.05
129.04
123.46
121.12
113.49
107.07
102.49
61.88
56.00
55.56
55.49
OMe
O
O
I
MeO
OMe
9
160 150 140 130 120 110 100 90 80 70 60 50 40 30 20 10 0
f1 (ppm)

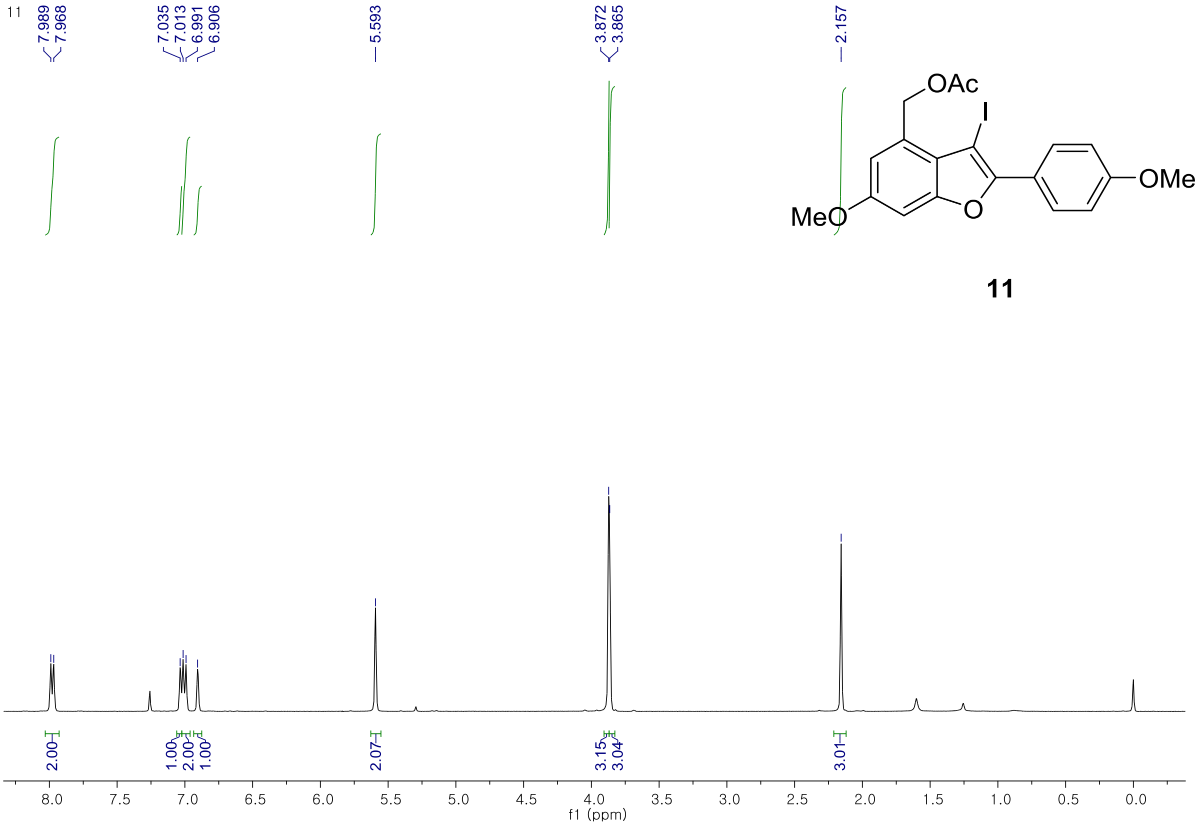

11
7.989
7.968
7.035
7.013
6.991
6.906
5.593
3.872
3.865
2.157
OAc
I
OMe
MeO
O
11
2.00
1.00
2.00
1.00
2.07
3.15
3.04
3.01
8.0 7.5 7.0 6.5 6.0 5.5 5.0 4.5 4.0 3.5 3.0 2.5 2.0 1.5 1.0 0.5 0.0
f1 (ppm)

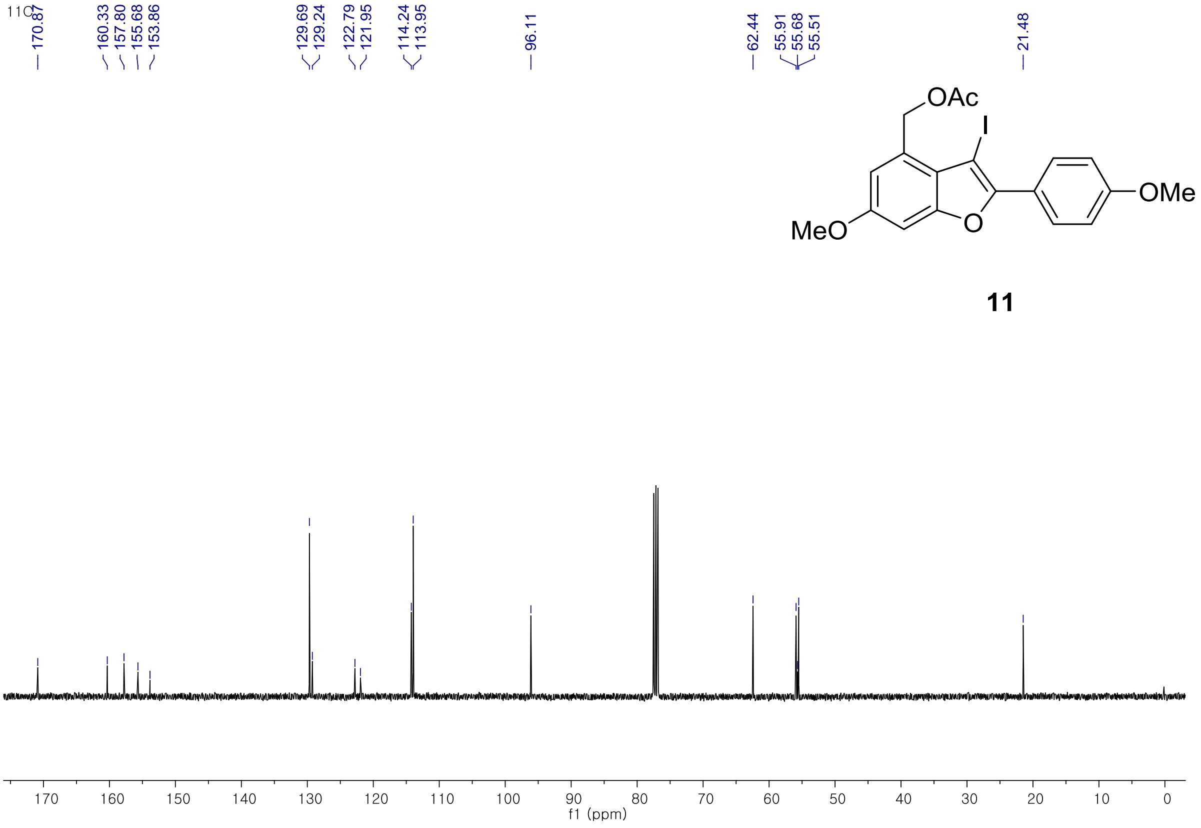

11C
170.87
160.33
157.80
155.68
153.86
129.69
129.24
122.79
121.95
114.24
113.95
96.11
62.44
55.91
55.68
55.51
21.48
OAc
I
OMe
MeO
O
11
170 160 150 140 130 120 110 100 90 80 70 60 50 40 30 20 10 0
f1 (ppm)

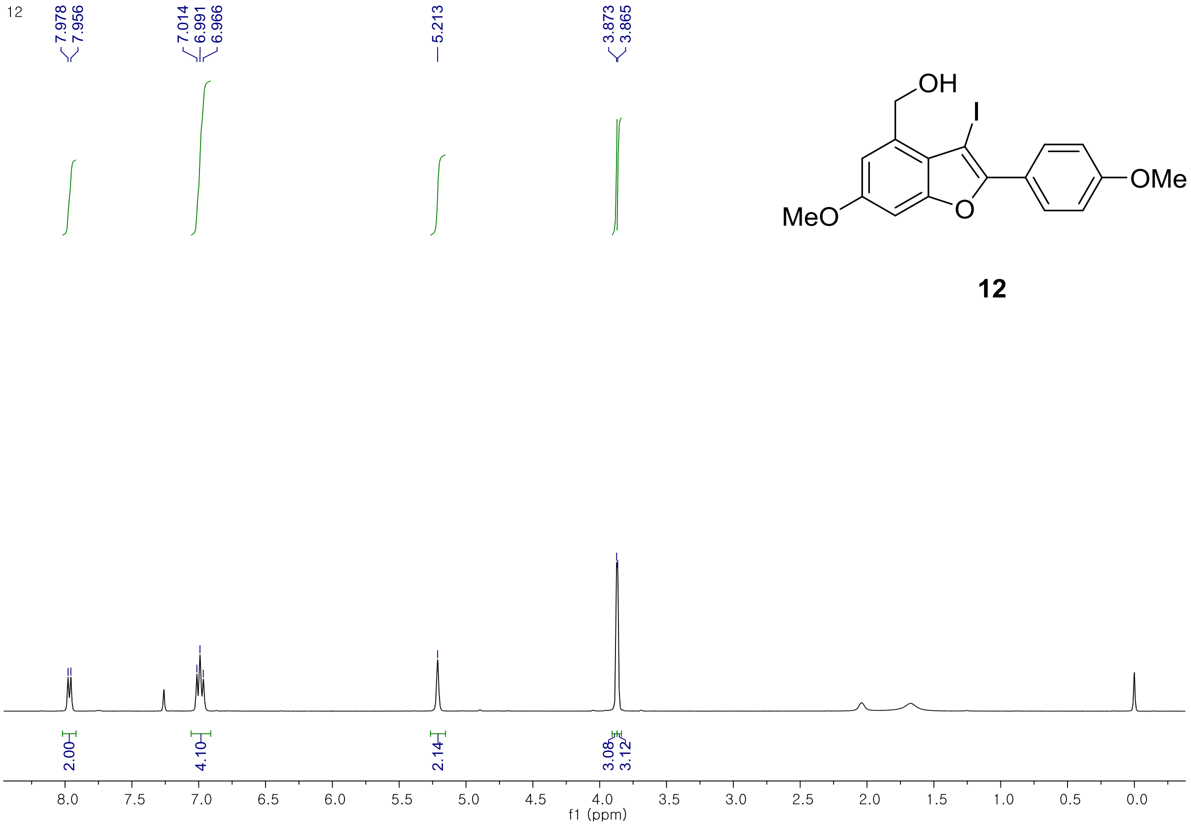
12
7.978
7.956
7.014
6.991
6.966
5.213
3.873
3.865
OH
I
OMe
MeO
O
12
2.00
4.10
2.14
3.08
3.12
f1 (ppm)

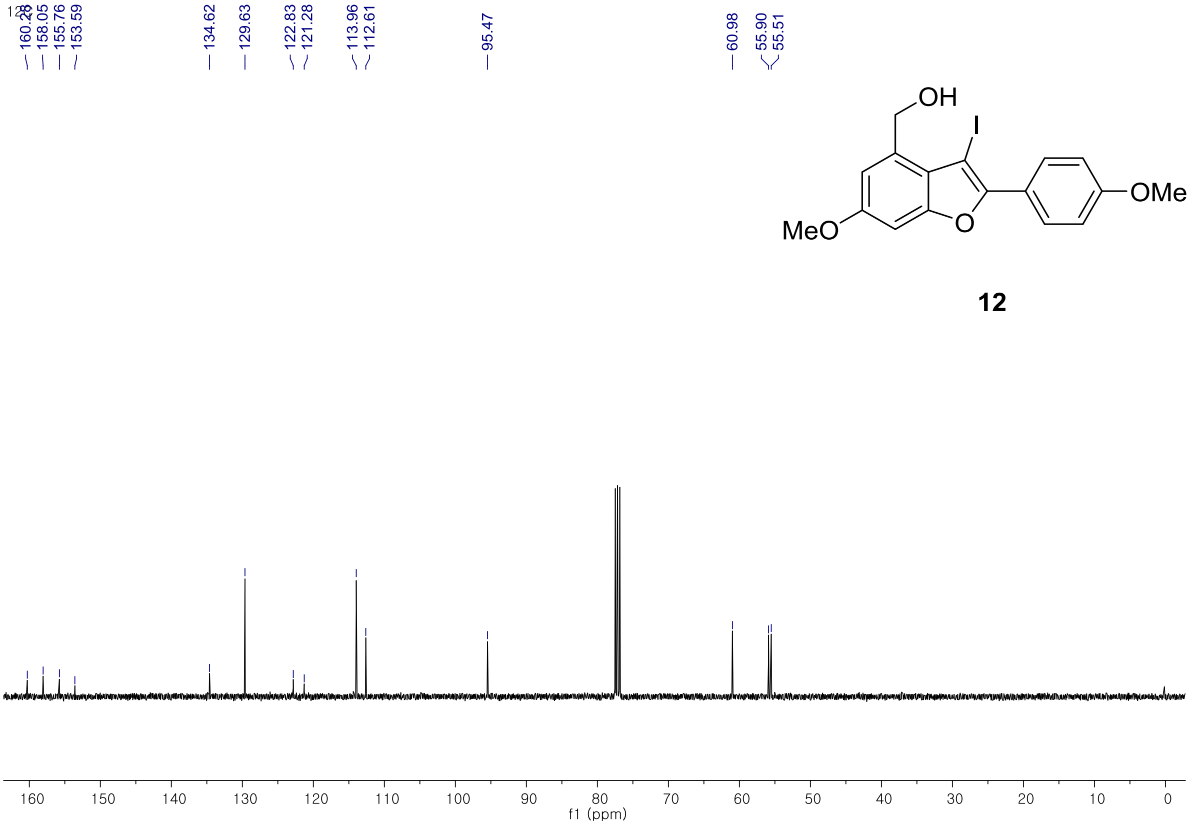
12
160.28
158.05
155.76
153.59
134.62
129.63
122.83
121.28
113.96
112.61
95.47
60.98
55.90
55.51
OH
I
OMe
MeO
O
12
f1 (ppm)

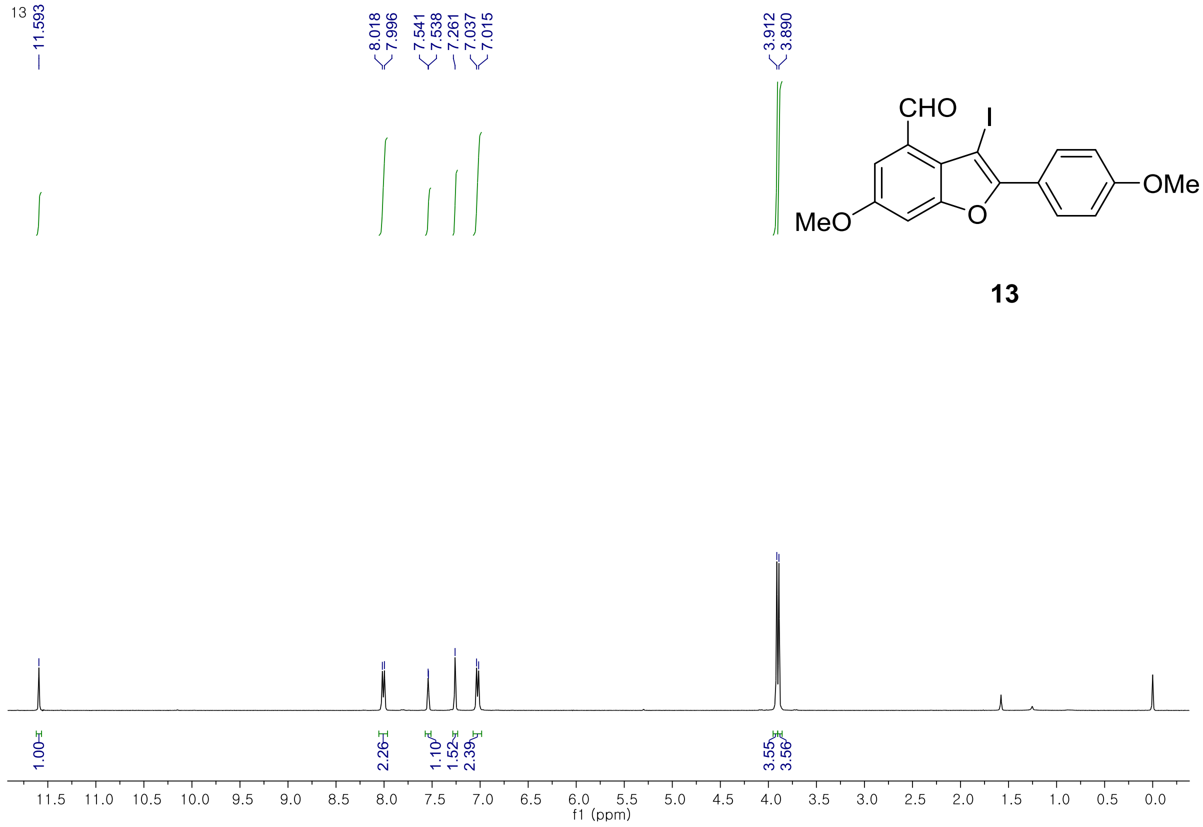
13
11.593
8.018
7.996
7.541
7.538
7.261
7.037
7.015
3.912
3.890
CHO
I
OMe
MeO
O
13
1.00
2.26
1.10
1.52
2.39
3.55
3.56
f1 (ppm)

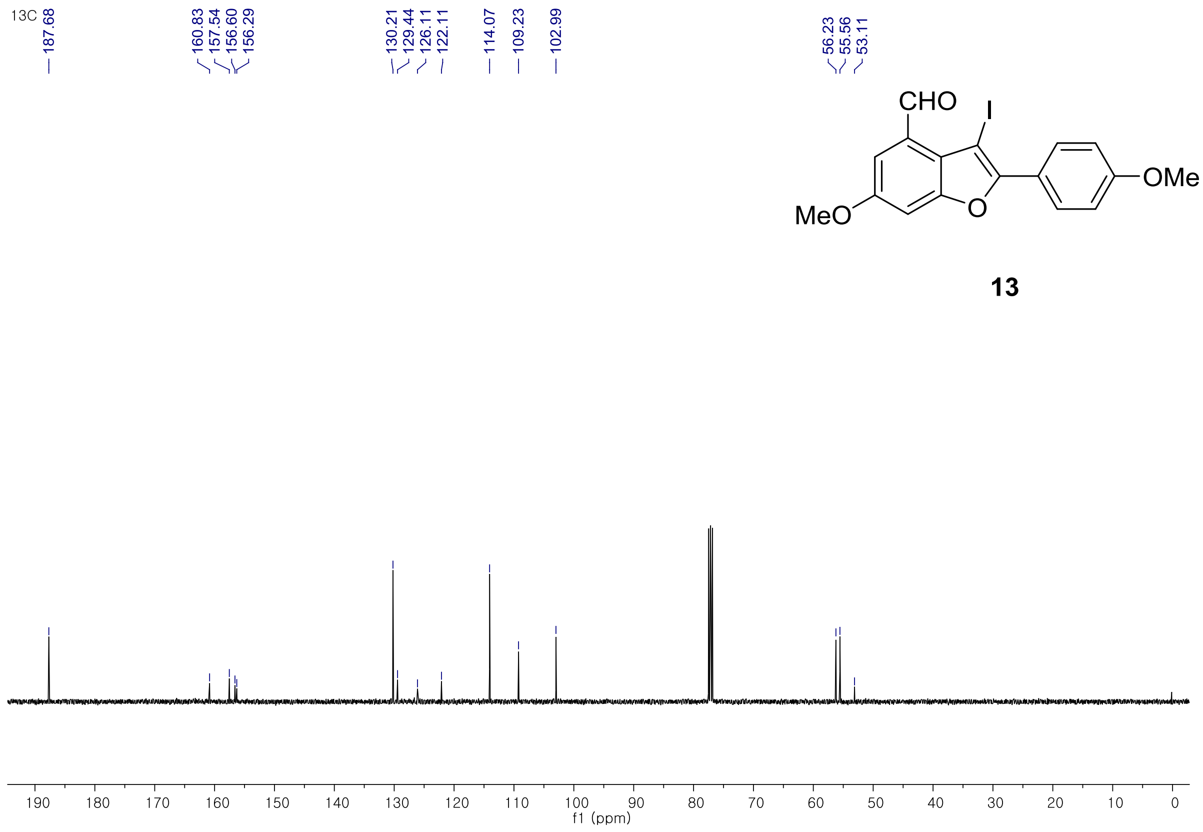
13C
187.68
160.83
157.54
156.60
156.29
130.21
129.44
126.11
122.11
114.07
109.23
102.99
56.23
55.56
53.11
CHO
I
OMe
MeO
O
13
f1 (ppm)

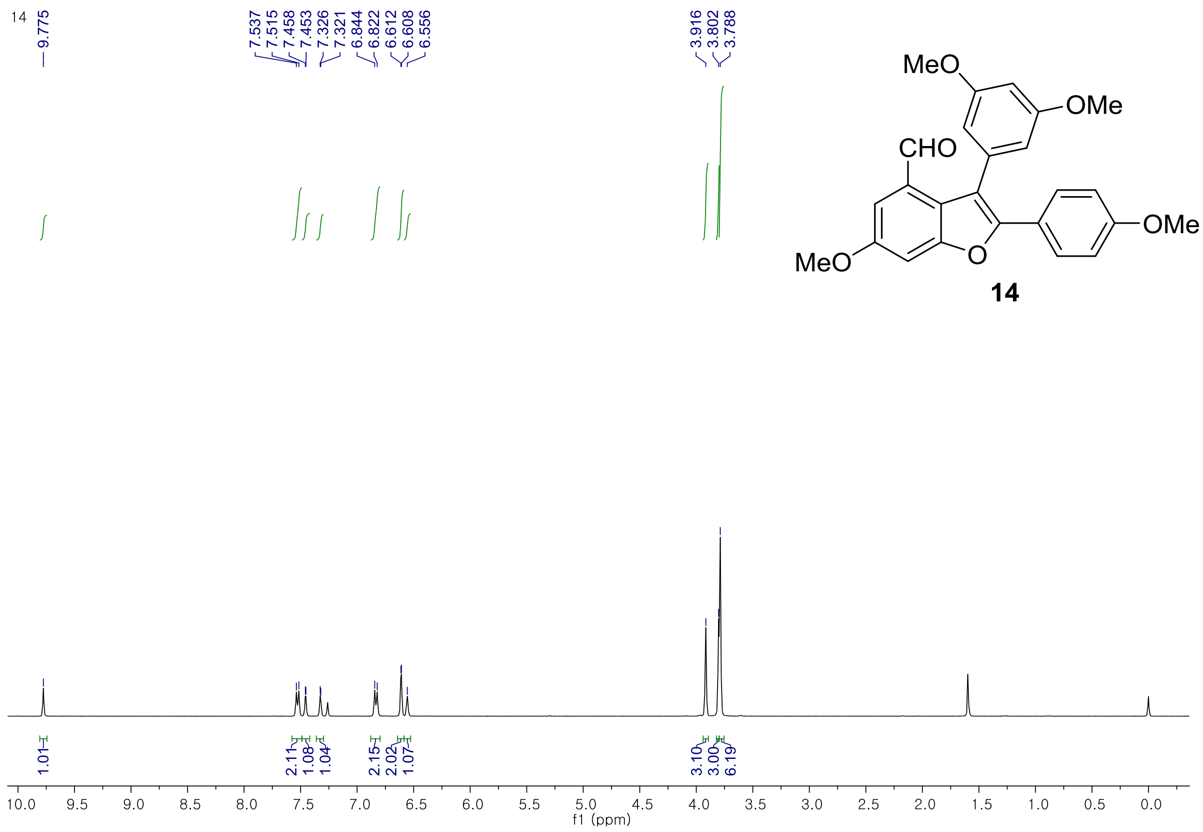

14
9.775
7.537
7.515
7.458
7.453
7.326
7.321
6.844
6.822
6.612
6.608
6.556
3.916
3.802
3.788
MeO
OMe
CHO
OMe
MeO
O
14
1.01
2.11
1.08
1.04
2.15
2.02
1.07
3.10
3.00
6.19
10.0 9.5 9.0 8.5 8.0 7.5 7.0 6.5 6.0 5.5 5.0 4.5 4.0 3.5 3.0 2.5 2.0 1.5 1.0 0.5 0.0
f1 (ppm)

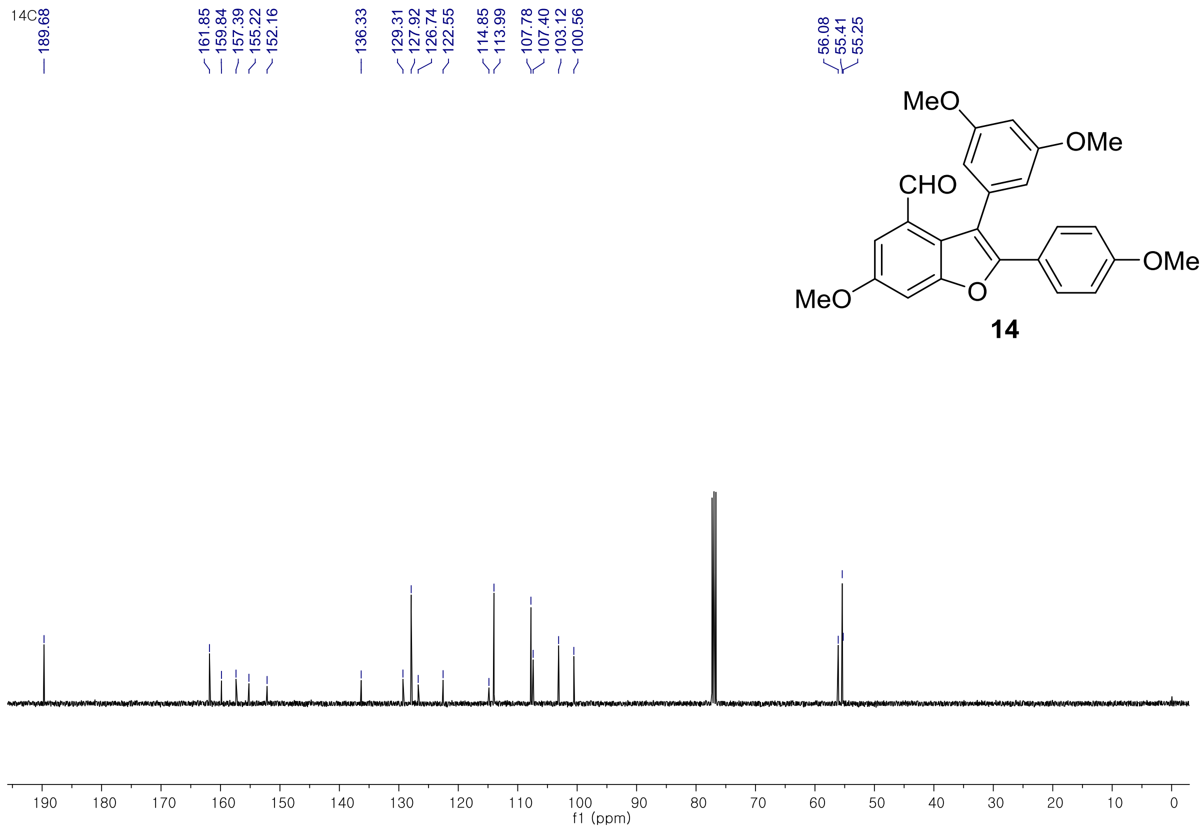

14C
189.68
161.85
159.84
157.39
155.22
152.16
136.33
129.31
127.92
126.74
122.55
114.85
113.99
107.78
107.40
103.12
100.56
56.08
55.41
55.25
MeO
OMe
CHO
OMe
MeO
O
14
190 180 170 160 150 140 130 120 110 100 90 80 70 60 50 40 30 20 10 0
f1 (ppm)

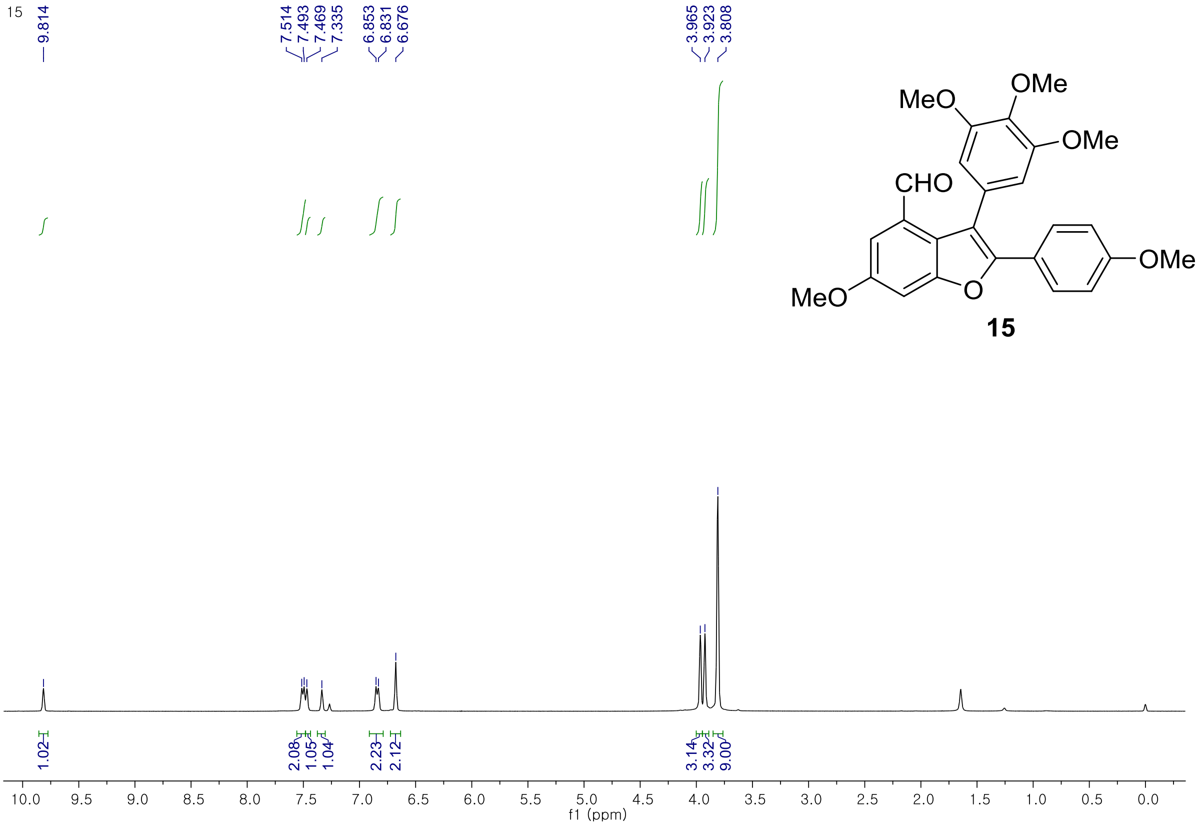
15
9.814
7.514
7.493
7.469
7.335
6.853
6.831
6.676
3.965
3.923
3.808
MeO
OMe
OMe
CHO
OMe
MeO
O
15
1.02
2.08
1.05
1.04
2.23
2.12
3.14
3.32
9.00
10.0 9.5 9.0 8.5 8.0 7.5 7.0 6.5 6.0 5.5 5.0 4.5 4.0 3.5 3.0 2.5 2.0 1.5 1.0 0.5 0.0
f1 (ppm)

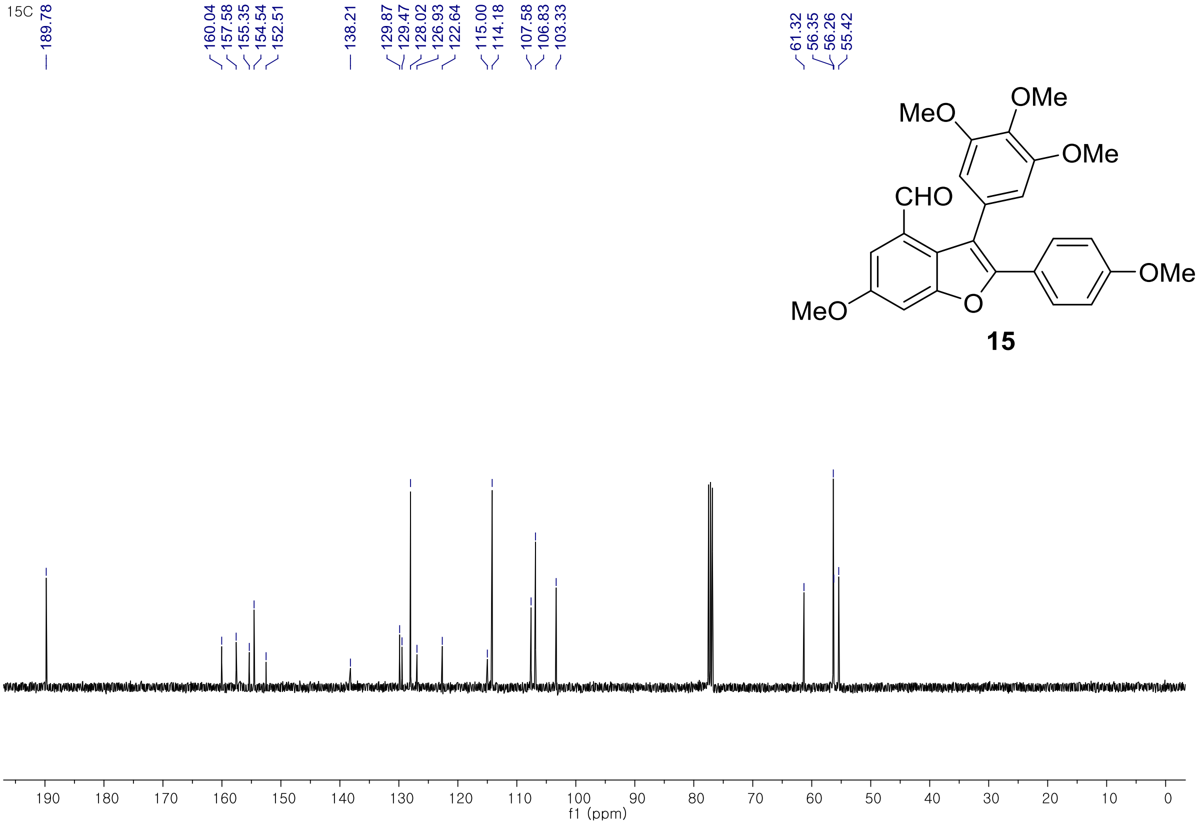
15C
189.78
160.04
157.58
155.35
154.54
152.51
138.21
129.87
129.47
128.02
126.93
122.64
115.00
114.18
107.58
106.83
103.33
61.32
56.35
56.26
55.42
MeO
OMe
OMe
CHO
OMe
MeO
O
15
190 180 170 160 150 140 130 120 110 100 90 80 70 60 50 40 30 20 10 0
f1 (ppm)

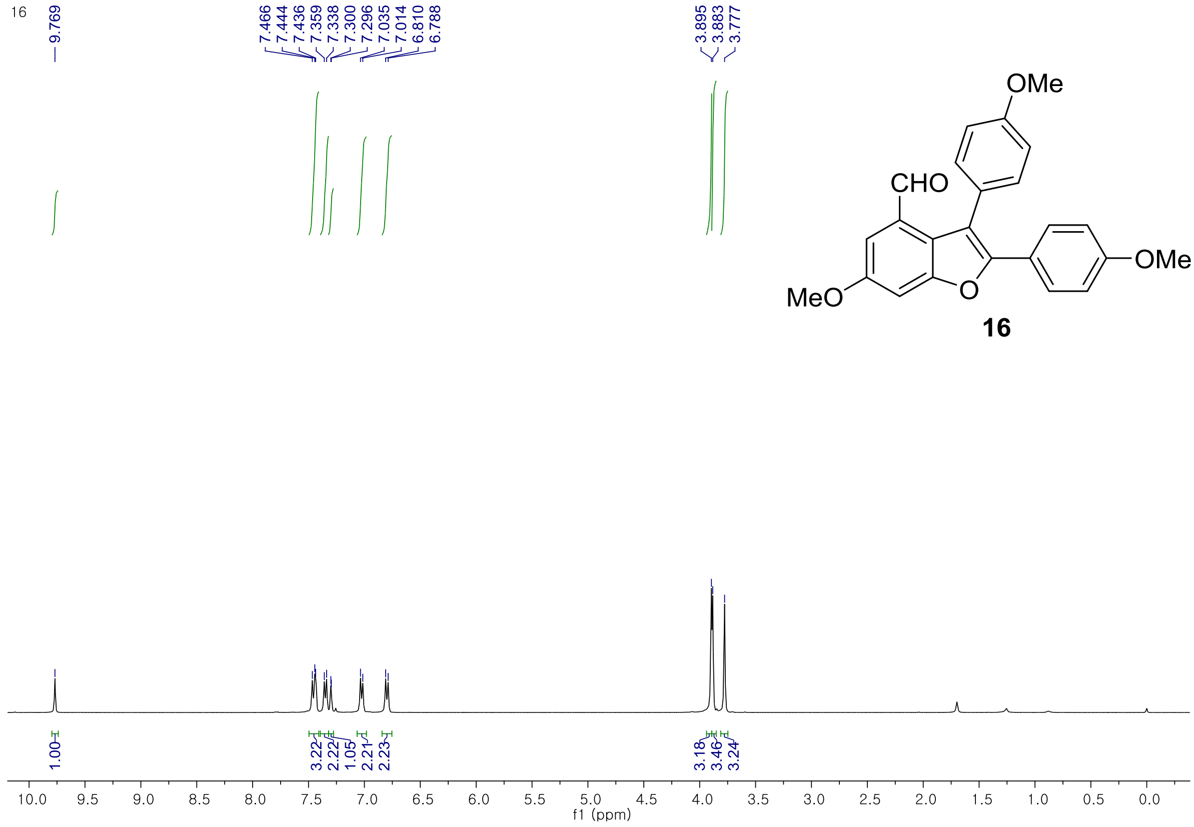
16
9.769
7.466
7.444
7.436
7.359
7.338
7.300
7.296
7.035
7.014
6.810
6.788
3.895
3.883
3.777
OMe
CHO
OMe
MeO
O
16
1.00
3.22
2.22
1.05
2.21
2.23
3.18
3.46
3.24
10.0 9.5 9.0 8.5 8.0 7.5 7.0 6.5 6.0 5.5 5.0 4.5 4.0 3.5 3.0 2.5 2.0 1.5 1.0 0.5 0.0
f1 (ppm)

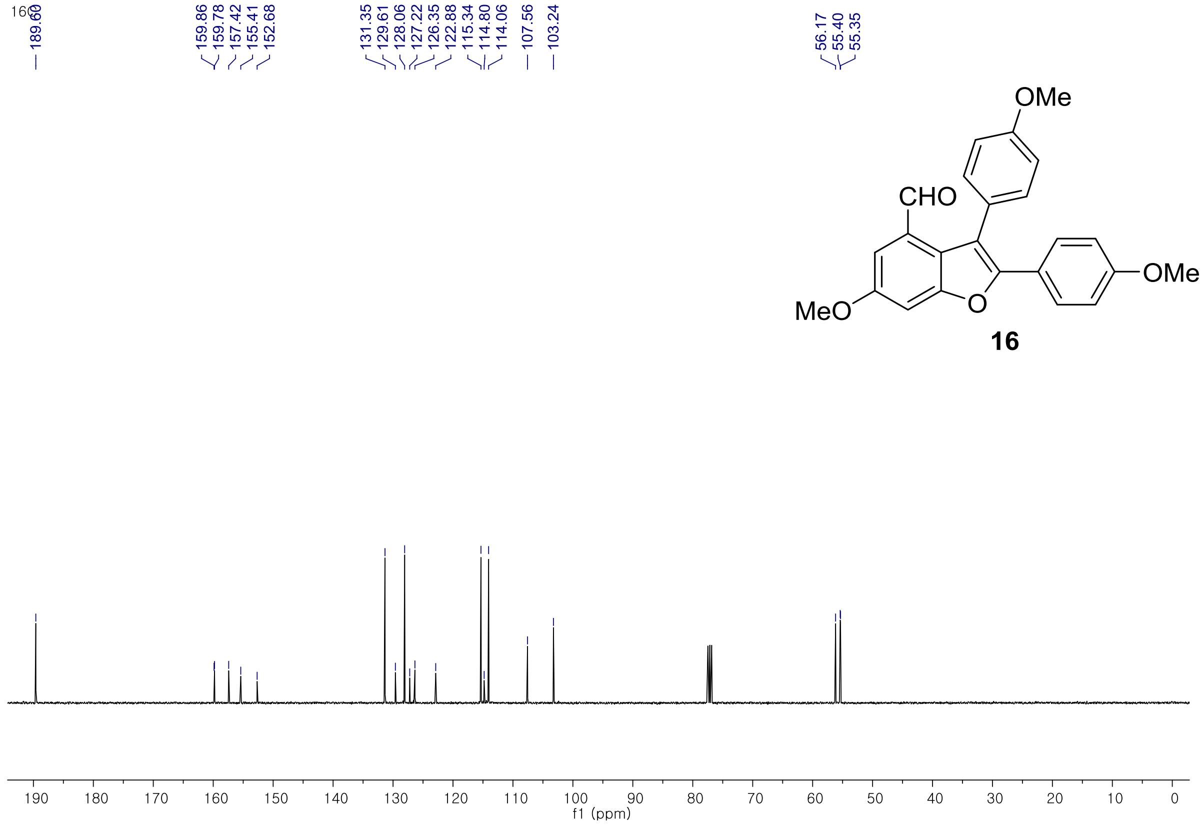
16
189.60
159.86
159.78
157.42
155.41
152.68
131.35
129.61
128.06
127.22
126.35
122.88
115.34
114.80
114.06
107.56
103.24
56.17
55.40
55.35
OMe
CHO
OMe
MeO
O
16
190 180 170 160 150 140 130 120 110 100 90 80 70 60 50 40 30 20 10 0
f1 (ppm)

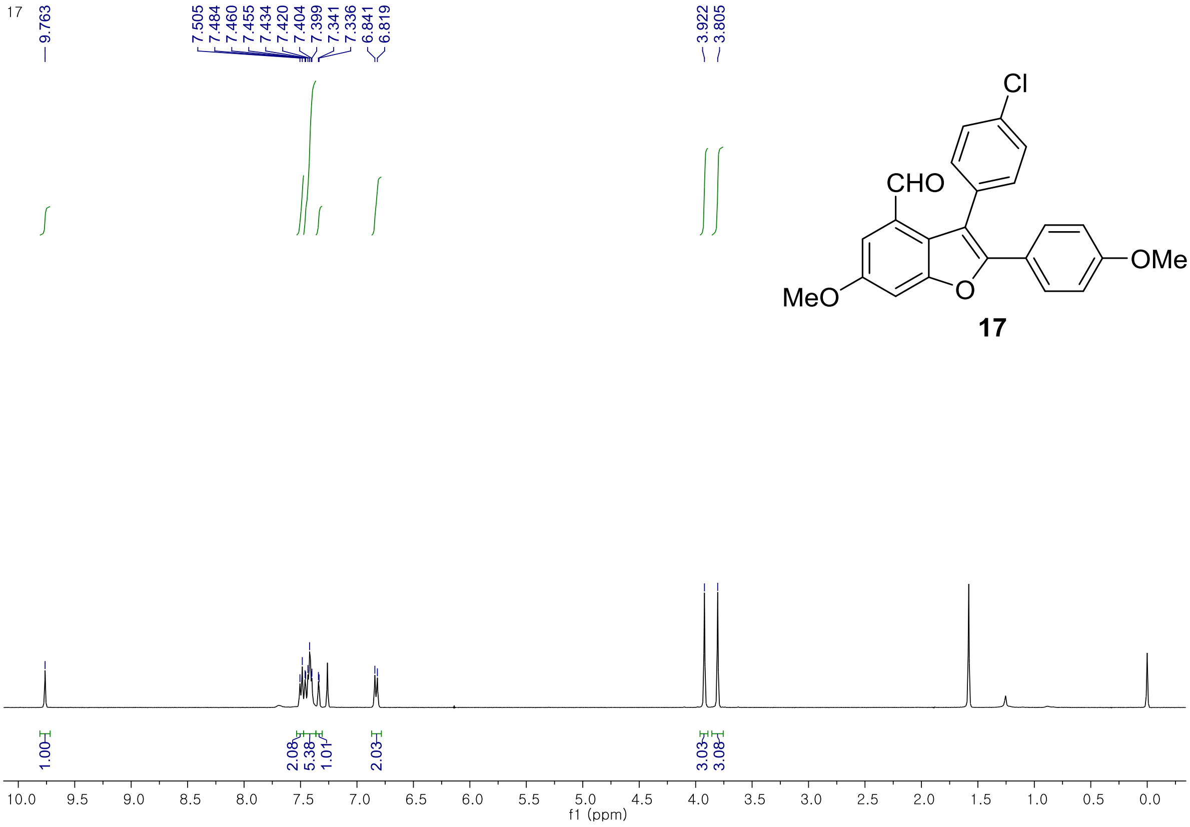
17
9.763
7.505
7.484
7.460
7.455
7.434
7.420
7.404
7.399
7.341
7.336
6.841
6.819
3.922
3.805
Cl
CHO
OMe
MeO
O
17
1.00
2.08
5.38
1.01
2.03
3.03
3.08
10.0 9.5 9.0 8.5 8.0 7.5 7.0 6.5 6.0 5.5 5.0 4.5 4.0 3.5 3.0 2.5 2.0 1.5 1.0 0.5 0.0
f1 (ppm)

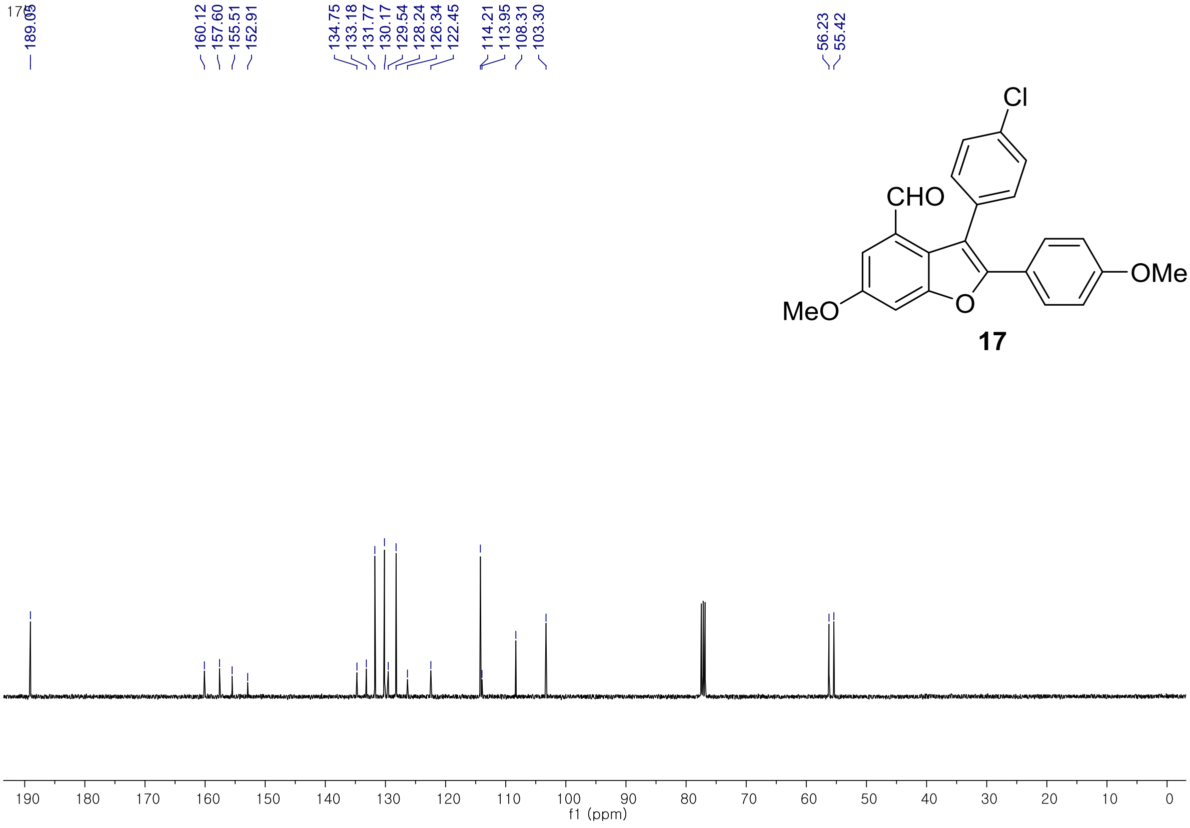
17
189.05
160.12
157.60
155.51
152.91
134.75
133.18
131.77
130.17
129.54
128.24
126.34
122.45
114.21
113.95
108.31
103.30
56.23
55.42
Cl
CHO
OMe
MeO
O
17
190 180 170 160 150 140 130 120 110 100 90 80 70 60 50 40 30 20 10 0
f1 (ppm)

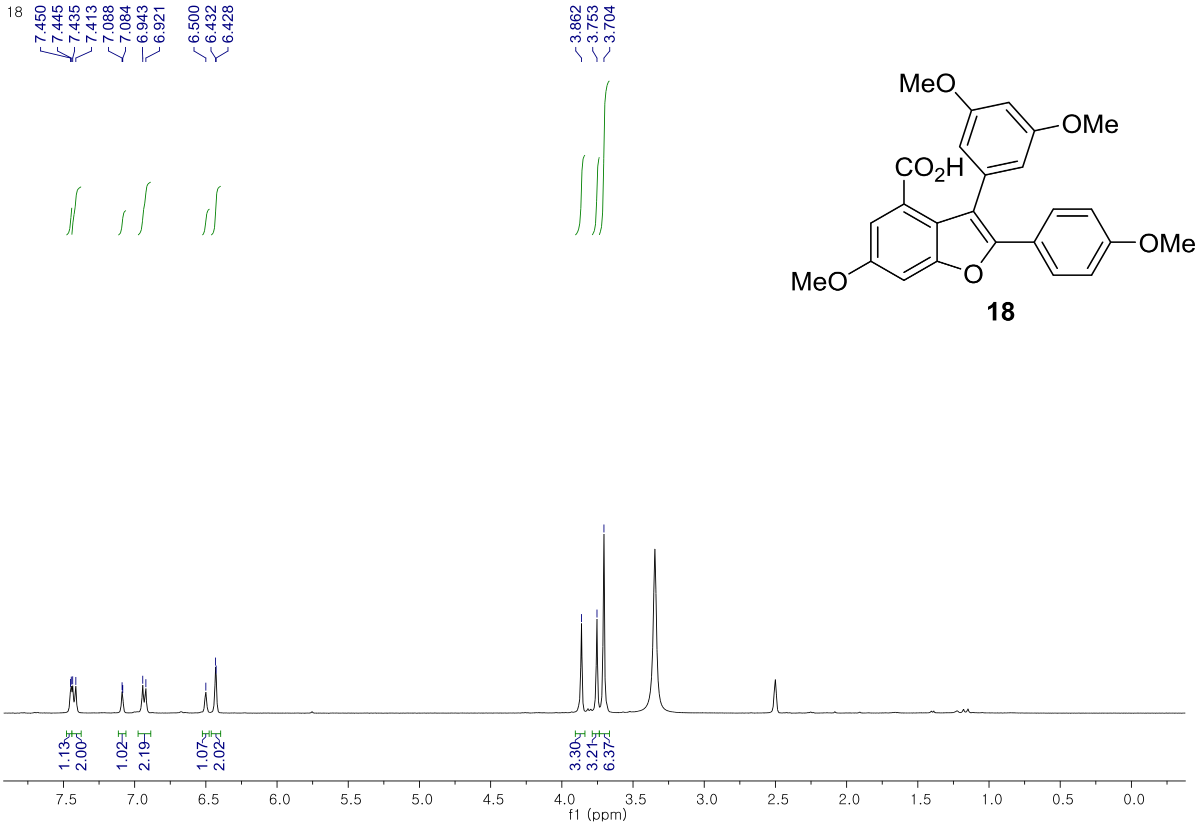
18
7.450
7.445
7.435
7.413
7.088
7.084
6.943
6.921
6.500
6.432
6.428
3.862
3.753
3.704
MeO
OMe
CO_2H
OMe
MeO
O
18
1.13
2.00
1.02
2.19
1.07
2.02
3.30
3.21
6.37
7.5 7.0 6.5 6.0 5.5 5.0 4.5 4.0 3.5 3.0 2.5 2.0 1.5 1.0 0.5 0.0
f1 (ppm)

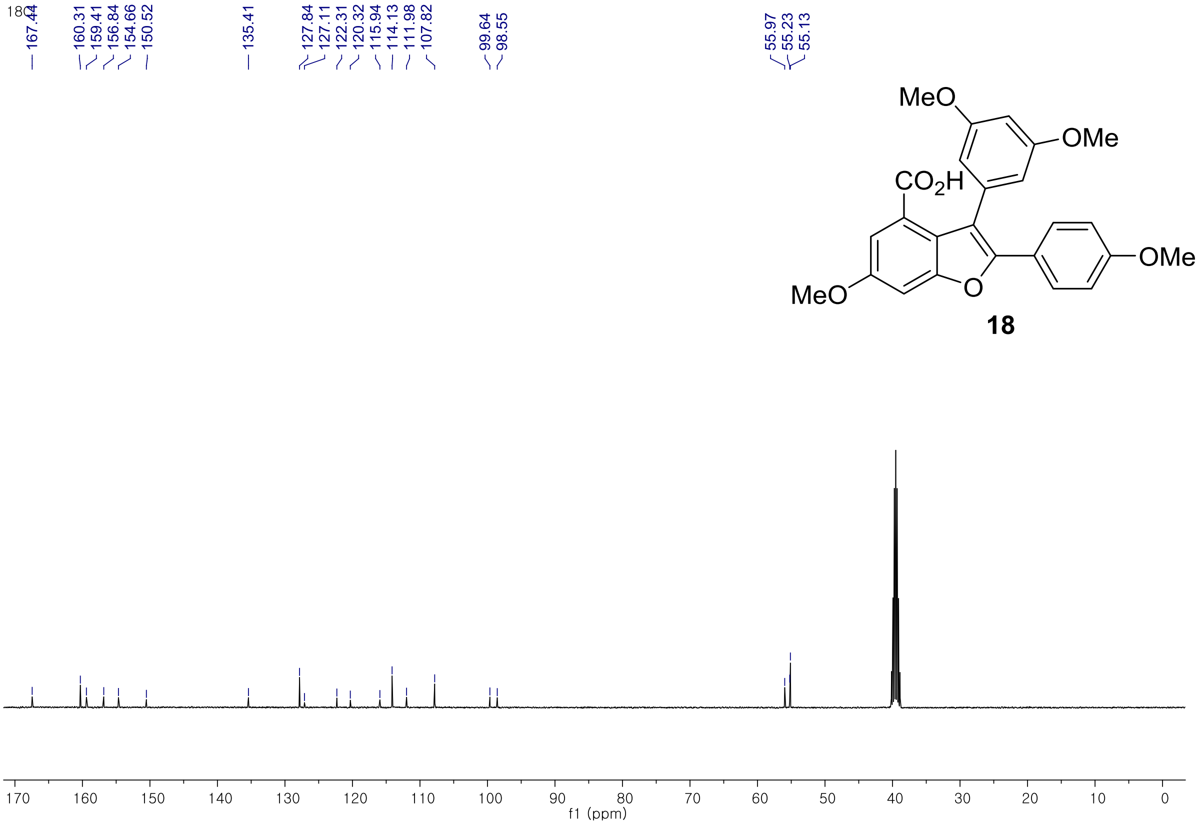
18C
167.44
160.31
159.41
156.84
154.66
150.52
135.41
127.84
127.11
122.31
120.32
115.94
114.13
111.98
107.82
99.64
98.55
55.97
55.23
55.13
MeO
OMe
CO_2H
OMe
MeO
O
18
170 160 150 140 130 120 110 100 90 80 70 60 50 40 30 20 10 0
f1 (ppm)
